# Choice of Differentiation Media Significantly Impacts Cell Lineage and Response to CFTR Modulators in Fully Differentiated Primary Cultures of Cystic Fibrosis Human Airway Epithelial Cells

**DOI:** 10.3390/cells9092137

**Published:** 2020-09-21

**Authors:** Vinciane Saint-Criq, Livia Delpiano, John Casement, Jennifer C. Onuora, JinHeng Lin, Michael A. Gray

**Affiliations:** 1 Biosciences Institute, University Medical School, Newcastle University, Framlington Place, Newcastle upon Tyne NE2 4HH, UK; vinciane.saint-criq@inrae.fr (V.S.-C.); L.Delpiano2@newcastle.ac.uk (L.D.); jennifer.c.onuora@gmail.com (J.C.O.); J.Lin21@newcastle.ac.uk (J.L.); 2 Bioinformatics Support Unit, Faculty of Medical Sciences, Newcastle University, Framlington Place, Newcastle upon Tyne NE2 4HH, UK; John.Casement@newcastle.ac.uk

**Keywords:** differentiation media, in vitro airway model, epithelium physiology, RNA-sequencing, pH, ion transport, cystic fibrosis, CFTR modulators

## Abstract

In vitro cultures of primary human airway epithelial cells (hAECs) grown at air–liquid interface have become a valuable tool to study airway biology under normal and pathologic conditions, and for drug discovery in lung diseases such as cystic fibrosis (CF). An increasing number of different differentiation media, are now available, making comparison of data between studies difficult. Here, we investigated the impact of two common differentiation media on phenotypic, transcriptomic, and physiological features of CF and non-CF epithelia. Cellular architecture and density were strongly impacted by the choice of medium. RNA-sequencing revealed a shift in airway cell lineage; one medium promoting differentiation into club and goblet cells whilst the other enriched the growth of ionocytes and multiciliated cells. Pathway analysis identified differential expression of genes involved in ion and fluid transport. Physiological assays (intracellular/extracellular pH, Ussing chamber) specifically showed that ATP12A and CFTR function were altered, impacting pH and transepithelial ion transport in CF hAECs. Importantly, the two media differentially affected functional responses to CFTR modulators. We argue that the effect of growth conditions should be appropriately determined depending on the scientific question and that our study can act as a guide for choosing the optimal growth medium for specific applications.

## 1. Introduction

In the respiratory tract, cells differentiate into over 40 different cell types with well-defined characteristics [1,2]. In addition to smooth muscle and endothelial cells, the most represented cell types are secretory cells (club and goblet cells), ciliated cells, neuroendocrine cells, and multipotent cells (basal cells). Recently, a rare and specialized cell subtype, known as the ionocyte, has been discovered [3,4,5]. The airways have been extensively characterized using ex vivo tissues, both in normal and chronic pathological situations (e.g., cancer, cystic fibrosis (CF), chronic obstructive pulmonary disease (COPD), and asthma), or after chemical insults. These types of in-depth studies require a large amount of freshly excised tissues that is not always available. Therefore, reconstituting an accurate, physiologically relevant in vitro human airway model is a powerful strategy to overcome this problem. While animals allow the study of physiological functions and systemic interactions between organs, in vitro models enable a more accurate and detailed exploration at the cellular and molecular level. These in vitro models also meet the principles of the 3 R’s (replacement, reduction, and refinement), reducing the use of animals, as well as having more biological relevance since they do not suffer from the species-specific differences encountered with animal models [6,7].

Primary airway cell cultures are developed from intact airways, nasal, tracheo-bronchial, or alveolar cells, and are usually obtained from explanted lungs, biopsies, or brushings [8]. The first airway cell cultures date back to the 1980s [9]; however, the process became more physiologically relevant with the introduction of growing cells at the air–liquid interface (ALI) [10], which greatly improved differentiation of the epithelial cultures that better matched the in vivo situation [11,12]. Cells grown at ALI develop a pseudostratified epithelium [13], consisting of more than one cell type (e.g., ciliated cells, goblet cells, and basal cells), which harbored tight junctions and generate distinct apical and basolateral membranes [10,14]. One of the major limitations of the in vitro airway models is the number of primary cells available, as well as their reduced proliferation/expansion capacity. This can be overcome by conditionally reprogramming the cells. In this case, primary cells are grown in the presence of a feeder (fibroblasts) layer and Rho kinase (ROCK) inhibitor, which enable the primary cells to proliferate indefinitely without losing their differentiation capacity [15,16]. Despite some limitations, this model allows complex cell culturing such as organoids [17] or co-cultures with immune cells [18], and also allows the use of the CRISPR gene editing [19], further increasing its importance as an in vitro model system. Several groups over the years have optimized airway cell extraction and growth conditions to help generate cell cultures that better mimic the in vivo situation [9,20,21,22,23,24], and this has led to an increase in the number of commercial companies offering their own bespoke expansion and differentiation media. In general, all the derived epithelia are characterized by a mucociliary phenotype displaying transepithelial Na^+^ absorption and Cl^−^ secretion, key ion transport mechanisms in airway epithelia.

The ALI cell culture method is now considered the gold standard for studies on airway biology under normal or pathologic conditions [25,26]. It is used for studies on inflammation [27], toxicology [28,29,30,31], immunology [32,33], drug discovery and delivery [34,35,36], and for personalized airway therapies [37]. Specifically, in CF research, the use of such differentiated ALI airway cultures has been instrumental in the development of CFTR (Cystic Fibrosis transmembrane conductance regulator)-directed therapies for people with CF [38,39] as well as in the identification of alternative non-CFTR therapeutic targets [40]. CF is a multiorgan monogenic autosomal recessive disease, in which lung pathology determines a patient’s life expectancy and quality of life [41]. It is caused by mutations of a single gene, CFTR, which is a chloride and bicarbonate channel that, in the airways, tightly regulates airway surface liquid (ASL) volume and composition [42]. CF airways are characterized by a dehydrated ASL [43], in which acid-base homeostasis is deregulated, and by the presence of a viscous mucus layer that provides a favorable environment for pathogen colonization [44].

One of the strengths of the ALI method is that it has been shown to be predictive of the outcomes following therapies [45]. However, there are numerous differentiation protocols in use today, which are used indiscriminately between research groups. This clearly underlines a lack of standardization of the existing ALI protocols that will undoubtedly affect reliability, reproducibility, and, importantly, the ability to compare results and conclusions between different studies. Recently, it was reported that the use of different culture media produced epithelia with different cell type proportions [46], and cells grown with different expansion media harbored distinct characteristics, especially regarding cell differentiation [47,48]. However, no studies to date have made an in-depth analysis of the effect of different growth media at both a transcriptomic and functional level. Finally, whether growth conditions impact on drug responses, and ultimately drug discovery, is currently unclear.

The aim of this study was therefore to characterize how two commonly used airway differentiation media affected the phenotypes of fully differentiated primary human airway epithelial cells (hAECs) from healthy (NCF for non-cystic fibrosis) and CF donors. Here, we show that although the epithelia displayed some degree of functional similarity, the choice of differentiation media profoundly affected histological, transcriptomic, and physiological features. Particularly relevant in the context of CF, the response to CFTR modulators was affected by growth conditions. This study provides an improved understanding of the specific characteristics of epithelia differentiated using different, but commonly used, growth conditions, and can act as a guide for choosing the optimal growth medium for a specific cell biological application.

## 2. Materials and Methods

### 2.1. Chemicals

Materials and chemicals were from Alfa-Aesar (Heysham, Lancashire, UK), Sigma-Aldrich (Gillingham, UK), Thermo Fisher Scientific (Gloucester, UK), CFF Therapeutics (Prof. R. Bridges’ lab, Rosalind Franklin University of Science and Medicine, Chicago, IL, USA) and Tocris Bioscence (Bristol, UK). See Table S1 in Supplementary Material.

#### Solutions

The following buffered solutions were used in the physiological assays: HCO_3_^−^ Krebs (HCO_3_^−^ KRB) contained, in mM: 25 NaHCO_3_, 115 NaCl, 5 KCl, 1 CaCl_2_, 1 MgCl_2_, 5 D-glucose, pH 7.4 at 37 °C, 5% CO_2_. Standard NaCl HEPES solution, hereafter called HEPES solution, contained, in mM: 130 NaCl, 5 KCl, 1 CaCl_2_, 1 MgCl_2_, 10 HEPES (sodium salt), 5 D-glucose, pH 7.4 at 37 °C. Zero K^+^ HCO_3_^−^ KRB solution, hereafter called 0K^+^ solution contained, in mM: 25 NaHCO_3_, 120 NaCl, 1 CaCl_2_, 1 MgCl_2_, 5 D-glucose. High K^+^ HEPES/Nigericin (10 μM final concentration) intracellular pH calibration solutions contained, in mM: 5 NaCl, 130 KCl, 1 CaCl_2_, 1 MgCl_2_, 10 NaHEPES, 5 D-glucose. ASL pH standard calibration solutions were modified Ringer solutions containing, in mM, 86 NaCl, 5 KCl, 1.2 CaCl_2_, 1.2 MgCl_2_, and either 50 MES (pH 5.5, 6.0 and 6.5), 50 HEPES (pH 7.0 and 7.5), or 50 Tris (pH 8.0) at 37 °C.

### 2.2. Cell Growth and Differentiation Protocol

Primary NCF (*n* = 3 donors) and CF (*n* = 3 donors, all F580del/F508del) human airway epithelial cells (hAECs) were a kind gift from Dr. Scott H. Randell (Marsico Lung Institute, The University of North Carolina at Chapel Hill, USA). The cells were obtained under protocol #03-1396 approved by the University of North Carolina at Chapel Hill Biomedical Institutional Review Board. Additional primary cells from 3 different CF donors (all F580del/F508del) were obtained via the CFFT Biorepository. Cells were expanded using the conditionally reprogrammed cell (CRC) culture method as previously described [15]. Briefly, cells were seeded on 3T3J2 fibroblasts inactivated with mitomycin C (4 µg/mL, 2 hr, 37 °C, M4287, Sigma-Aldrich) and grown in medium containing the ROCK inhibitor Y-27632 (10 μM, Tocris Bioscence) until they reached 80% confluence. Cells then underwent double trypsinization to first remove the fibroblasts and to then detach the hAECs from the P150 dish. At that stage, cells were counted and frozen down in 89% Ham’s F12 medium, 5% FBS (fetal bovine serum), 5% DMSO (Sigma-Aldrich), 1% 1.5 M HEPES (Sigma-Aldrich).

**Figure 1:**
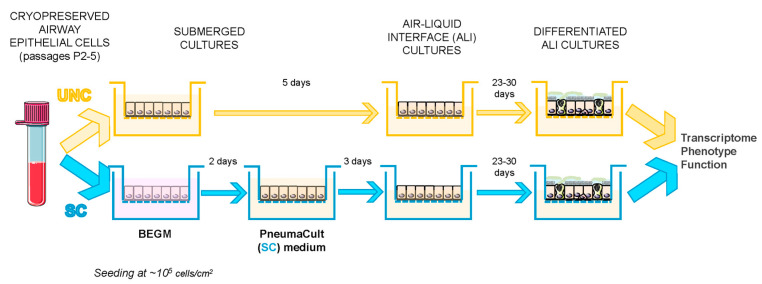
General overview of the protocol. Schematic representation of the workflow used to differentiate primary human airway epithelial cells using the UNC or SC differentiation media. Human airway epithelial cells (hAECs) preserved in liquid nitrogen were thawed and seeded at the same density (10^5^/cm^2^) in either UNC or BEGM with media in both apical and basolateral compartments. After 2 days, cells seeded in BEGM were switched to SC medium bilaterally and 5 days after seeding, apical medium was removed in order to generate an air–liquid interface (ALI). Differentiation was allowed to occur for 23 to 30 days, after which, the cells were then used for phenotypic, transcriptomic, and functional analyses.

The protocol for comparing the effect of the differentiation media is presented in Figure 1 and was as follows: cryopreserved cells were seeded onto semi-permeable supports (Costar 6.5 or 12 mm, Sigma-Aldrich) either in bilateral differentiating medium previously described by Randell et al. [49], hereafter called UNC, or in bilateral BEGM (composition of these media can be found in the Supplementary Table S2). For the latter condition, after 2 days, BEGM medium was replaced bilaterally by a commercially available medium, hereafter referred to as SC (StemCell PneumaCult™-ALI Medium, Catalog #05001, STEMCELL Technologies, Cambridge, UK, prepared according to the manufacturer’s instructions). After 5 days for the UNC condition, and a further 3 days for the SC condition (a total of 5 days after seeding), apical medium was removed to allow the cells to differentiate under ALI conditions. Cells were fed three times a week. Ciliogenesis started approximately 12–15 days after seeding and cells were used for experiments between days 28 and 35 after seeding (23 to 30 after ALI). Cells seeded on 12-mm supports were used for either RNA extraction to perform transcriptomic studies (RNA-sequencing and RT-qPCR), protein extraction, or for intracellular pH (pH_i_) measurements, whereas cells seeded on 6.5-mm transwells were used for phenotypic analysis (histology and immunofluorescence), ion transport measurements in Ussing chambers, and airway surface liquid (ASL) pH measurements.

In some experiments, differentiated CF epithelial cells were additionally treated for 48 h with the CFTR corrector VX-809 (3 μM, basolateral) or vehicle control (DMSO 0.1 % *v*/*v*).

### 2.3. RNA Extraction, RNA-Sequencing, and Real-Time Quantitative PCR Analysis

RNA isolation from fully differentiated hAECs was performed using the PureLink^®^ RNA Mini Kit (12183018A, Ambion, Life Technologies, Warrington, UK), following the manufacturer’s instructions. Briefly, lysates were mixed with 70% ethanol and loaded onto a silica-membrane column. Columns were washed and total RNA was eluted in nuclease-free water and stored at −80 °C until use.

The quality of all samples was assessed with a TapeStation 4200 (Agilent Technologies, Santa Clara, CA, USA) to verify RNA integrity. All samples had an RNA integrity number >8. Sequencing libraries were prepared using a TruSeq stranded mRNA sample preparation kit (Illumina, San Diego, CA, USA) following manufacturer’s instructions. Pooled libraries were sequenced at >15 million (75 bp) single reads per sample using a NextSeq 500 and high output kit (75 cycles) (Illumina). The RNA-sequencing data are accessible through Gene Expression Omnibus (GEO) series accession number GSE154905.

Single gene expression via RT-qPCR (real-time quantitative PCR) was also performed to further validate results from the RNA-sequencing (RNA-seq) data. Prior to reverse transcription, DNase treatment was performed on 300 ng RNA using RNAse-free DNAse I (04716728001, Roche, Basel, Switzerland) at 37 °C for 10 min. Reaction was then stopped by increasing the temperature to 70 °C for 10 min. Complementary DNA (cDNA) was synthesized from DNase I-treated total RNA (300 ng) using M-MLV reverse transcriptase (M1705, Promega, Southampton, UK) as per supplier’s protocol (1 h at 37 °C followed by 10 min at 70 °C). Real-time quantitative PCR (qPCR) was performed in a total volume of 15 µL using 2× LightCycler^®^ 480 SYBR Green I Master (04707516001, Roche, Basel, Switzerland), 1.5 µL of cDNA, 0.2 μM forward primer, and 0.2 μM reverse primer (primers were from Integrated DNA Technologies, Leuven, Belgium) in a 96-well plate (Table 1). PCR was run with the standard program: 95 °C for 10 min, 40 times of cycling at 95 °C for 15 s, and 60 °C for 1 min in a 96-well plate. Results are shown as relative quantity (RQ) of mRNA copies to the determined control condition (UNC), calculated using the 2^−ΔΔCt^ method and using 18S rRNA as a housekeeping gene.

### 2.4. Protein Extraction, Quantification, and Western Blot (WB)

Differentiated epithelia were washed twice with cold PBS and then lysed in RIPA (Radioimmunoprecipitation assay) buffer containing proteases inhibitors. The extracted proteins were quantified with BCA protein assay (10678484, Thermo Fisher Scientific) according to the manufacturer’s protocol. ATP12A and CFTR protein levels were evaluated by Western blot (WB) analysis. Thirty µl of proteins (containing 30 µg of proteins) were incubated at 37 °C for 15 min and then loaded onto 8% SDS-PAGE gels. Proteins were electrophoretically separated and transferred onto activated PVDF membranes using a semi-dry transfer method (Trans-Blot Turbo Transfer system, Bio-Rad, Watford, UK). Membranes were blocked for one hour at room temperature with 5% skimmed milk in TBS-T (Tris-Buffered Saline-Tween, 50 mM Tris pH 7.4, 150 mM NaCl, 0.1% TWEEN-20) and further incubated with primary antibodies against CFTR, ATP12A, or β-actin (in TBS-T with 5% skimmed milk, Table 2) overnight at 4 °C. The membranes were then washed three times at RT with TBS-T and the HRP-conjugated secondary antibodies (in TBS-T with 5% skimmed milk, Table 2) were added onto the membranes and incubated at room temperature for 1 h. Membranes were then washed three times with TBS-T and Western blots were developed using ECL (enhanced chemiluminescence reagents) according to the manufacturer’s instructions. Images were obtained and processed by using a luminescent image analyzer (LAS4000; Fujifilm, Cambridge, MA, USA). Band detection and densitometry were performed using Fiji imaging software [50].

### 2.5. Histological and Immunofluorescence Staining

Cells were washed 3 times with ice-cold PBS (Phosphate-buffered saline, Thermo Fisher Scientific). After the last wash, 4% paraformaldehyde (PFA, Sigma-Aldrich), reconstituted in sterile PBS and filtered, was added to both apical and basolateral compartments and incubated for 20 min at room temperature. PFA was then removed and cells were further washed 3 times with PBS. Fixed cells were then stored at 4 °C in PBS until use.

#### 2.5.1. Histology

Fixed cells on semi-permeable supports were detached from the plastic holder using a scalpel, carefully cut in 2, and placed in a histological cassette kept in 70% EtOH, followed by exposure to solutions of increasing EtOH concentration, to allow for sample dehydration. Epithelia were further processed and embedded in paraffin, blocks were cut (5-µm thick) with a rotating microtome and sections collected onto slides (cat. #10149870, Thermo Fisher Scientific, SuperFrost Plus Adhesion slides). Slides were then incubated at 60 °C for 2 h to allow for complete adhesion and removal of extra paraffin. They were then rehydrated by sequential immersion in xylene (534056, Sigma-Aldrich) for 5 min and then for 1 min in solutions of decreasing EtOH concentrations (99 to 70%) and finally rinsed with dH_2_O. Alcian blue-periodic acid–Schiff (AB-PAS) staining was then performed by incubating the slides for 5 min in Alcian blue (B8438, Sigma-Aldrich), periodic acid solution (Reagent 1 of the PAS staining kit; 1016460001, Sigma-Aldrich) for 10 min and Schiff’s reagent (Reagent 2 of the PAS staining kit) for 15 min (slides were rinsed in running water and then dH_2_0 between each incubation). Following this, slides were dehydrated by sequential incubation in solutions containing increasing ethanol concentrations and cleared in xylene for 5 min before being mounted using DPX mountant (06522, Sigma-Aldrich) and a 22-mm square coverslip. Slides were left to dry overnight before imaging using a Zeiss Axio Imager 2 (Cambridge, UK). Epithelial thickness was later measured using Fiji imaging software [50].

#### 2.5.2. Immunofluorescence Staining

**Figure 2:**
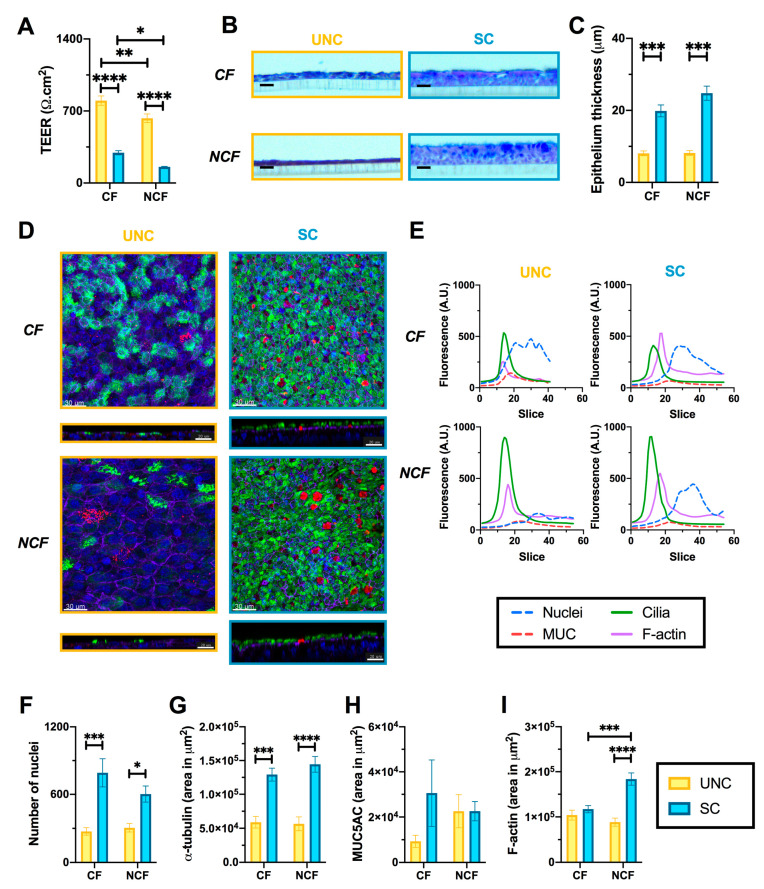
UNC and SC media induce distinct epithelial phenotypes. (**A**) Transepithelial electrical resistance of differentiated CF and non-CF (NCF) epithelia grown in UNC or SC. Differentiated cells were further fixed and processed in order to perform histological (HC, (**B**,**C**)) and immunofluorescent staining (IF, (**D**–**F**)) analyses. (**B**) Representative images of CF and NCF hAECs grown in UNC and SC media which were fixed, embedded in paraffin, sectioned, and stained with Alcian blue/periodic acid–Schiff reagents (scale bar is 20 μM). (**C**) Epithelium thickness (in μM) of UNC- and SC-grown CF and NCF cells measured from fixed HC slices. (**D**) Representative images of CF (top panels) and NCF (bottom panels) cells grown in UNC (left panels) and SC (right panels) stained with anti-α-tubulin (green), anti-MUC5AC (red), phalloidin (purple), and DAPI (blue) allowed for fluorescence profiling along the z-stack (**E**), quantification of the number of nuclei (**F**), α-tubulin (**G**), MUC5AC (**H**), and F-actin (**I**). Scale bars on D are 30 μM for XY images and 20 μM for XZ images. For all graphs, UNC-grown cells are in yellow, SC-grown cells in blue, bargraphs and error bars represent mean ± sem, 2-way ANOVA with Sidak’s multiple comparisons test; * *p* < 0.05, ** *p* < 0.01, *** *p* < 0.001, **** *p* < 0.0001.

PFA-fixed epithelia were washed with PBS for 5 min at RT with gentle shaking before being permeabilized using a solution of 0.3% Triton in PBS (5 min, RT, Sigma-Aldrich) washed again for 5 min with PBS, blocked in 5% BSA in PBS (1 h, RT), washed for 5 min in PBS, and incubated over night at 4 °C with the primary antibodies diluted in 1% BSA in PBS (Table 2). The inserts were then washed 3 times for 5 min in PBS and incubated in the dark with the secondary antibodies (Table 2) together with Phalloidin-Alexa Fluor 647 (A30107, Thermo Fisher Scientific, for 1 h at room temperature. DAPI (in PBS) was added for 5 min before membranes were further washed (5 min, PBS), cut from the plastic support, and attached to the bottom of a 60-mm dish using transparent nail polish. Imaging was performed using a Nikon A1plus Confocal Microscope (pinhole size 33.21 µm, x-y image size 196.30 × 196.30 µm (1024 × 1024 pixels), z-step size 0.72 µm, Nikon U.K. Ltd. Kingston upon Thames, UK). Between 50 and 100 stacks were acquired per sample, with the four channels acquiring sequentially to minimize spectral overlap. Confocal stacks were imported into the Imaris x64 9.3.0 software (Bitplane, Oxford Instruments, Abingdon, UK) where a semi-automated analysis of the different cell structures was performed. For each image, the results reported the number of surfaces detected and their area that was combined. Additionally, the fluorescence signals through the z-stacks, shown in Figure 2E, were measured using Fiji software.

### 2.6. Intracellular pH

Primary airway epithelial cells grown on 12-mm supports were loaded with the pH-sensitive, fluorescent dye BCECF-AM (10 µM) for 1 h in a HEPES solution at 37 °C. Cells were mounted onto the stage of a Nikon fluor inverted microscope and perfused with a modified HCO_3_^−^ KRB solution gassed with 5% (*v*/*v*) CO_2_/95% (*v*/*v*) O_2_. Solutions at 37 °C were perfused across the apical and basolateral surfaces at a flow rate of 3 mL.min^−1^ (0.5 mL total volume) and 6 mL.min^−1^ (1.0 mL total volume), respectively. Intracellular pH (pH_i_) was measured using a Life Sciences microfluorimeter system (Life Sciences Resources, Cambridge, UK) in which cells were alternately excited at 490 and 440 nm wavelengths every 1.024 s with emitted light collected at 510 nm. The ratio of 490 to 440 nm emission was recorded using PhoCal 1.6 b software (Life Sciences Resources, Cambridge, UK) and calibrated to pH_i_ using the high K^+^/nigericin technique [51] in which cells were exposed to high K^+^ solutions containing 10 µM nigericin (N7143, Sigma-Aldrich), set to a desired pH, ranging from 6 to 7.5. For analysis of pH_i_ measurements, ΔpH_i_ was determined by calculating the mean pH_i_ over 60 s resulting from treatment. The initial rate of pH_i_ change (ΔpH_i_/Δt) was determined by performing a linear regression over a period of at least 40 s.

### 2.7. Short-Circuit Current Measurements in Ussing Chamber

Cells grown on 6.5 mm-inserts were mounted into the EasyMount Ussing Chamber Systems (VCC MC8 Physiologic Instrument) and bathed in bilateral HCO_3_^−^KRB continuously gassed with 95% O_2−_ 5% CO_2_ and maintained at 37 °C. Monolayers were voltage-clamped to 0 mV and cells were left to equilibrate for at least 20 min before ion transport agonists and inhibitors were added. These were added in the following sequence: amiloride (10 µM, apical; A7410, Sigma-Aldrich), forskolin (Fsk, 10 µM, bilateral; 1099, Tocris Bioscience), potentiator 5, P5 (dF508act-02, 5 µM, apical; CFF Therapeutics, CFTR Compound Program), and CFTR_inh172_ (172, 20 µM, apical; 3430, Tocris Bioscience). Changes in short-circuit current (ΔIsc) were monitored using Ag/AgCl reference electrodes. The transepithelial short-circuit current (Isc) and the transepithelial electrical resistance (TEER) were recorded using Ag-AgCl electrodes in 3 M KCl agar bridges and the Acquire & Analyze software (Physiologic Instruments, San Diego, CA, USA), as previously described [52], and results normalized to an area of 1 cm^2^ and expressed as Isc (µAmp.cm^−2^).

### 2.8. ASL pH

Cells grown on 6.5-mm transwells were washed apically with 120 µL glucose-free HCO_3_^−^KRB for 15 min at 37 °C, 5% CO_2_. The ASL was stained using 3 µL of a mixture of dextran-coupled pH-sensitive pHrodo Red (0.5 mg/mL, λex: 565 nm, λem: 585 nm, P10361, ThermoFisher Scientific) and dextran-coupled pH-insensitive Alexa Fluor^®^ 488 (0.5 mg/mL, λex: 495 nm, λem: 519 nm, D22910, ThermoFisher Scientific) diluted in glucose-free HCO_3_^−^ KRB, overnight at 37 °C, 5% CO_2_. Alexa Fluor^®^ 488 was used as a loading control as pHrodo is not a ratiometric dye. The next day, fluorescence was recorded using a temperature and CO_2_-controlled plate reader (TECAN SPARK 10M, Tecan UK, Theale, UK) and agonists (Forskolin and P5) were added to the basolateral compartment at the indicated times (see figures). The data analysis was performed as previously described [53]: after subtracting background values from pHrodo and Alexa Fluor^®^ 488, ratios were generated for each time point and pH was calculated from a standard curve where pH was clamped using highly buffered solutions between 5.5 and 8. To reduce inter-experiment variability, the standard curve calibration was performed at the end of each independent experiment.

### 2.9. Data Analysis

#### 2.9.1. RNA-Sequencing Analysis

Quality of FASTQ files was assessed with FastQC, Version 11.7. No read trimming was necessary, and all samples were retained for further analysis. Reads were quantified against transcripts using Salmon, a program for quantifying expression of transcripts from RNA-Seq data. Salmon quantifies reads against transcripts. To obtain gene-level counts, the R package ‘tximport’ was used. Gene annotation was obtained from Ensembl gene ids using the R package ‘biomaRt’. Differential expression analysis was carried out using the R package DESeq2 [54]. DESeq2 estimates fold change between experimental conditions using a Negative Binomial GLM with a logarithmic link function. The resulting estimates of logarithmic fold change (Log_2_FC) were tested for significance using a Wald test, in which the estimate of Log_2_FC was divided by its standard error to obtain a test statistic, which was then compared to a standard normal distribution.

#### 2.9.2. Airway Epithelial Cell Subtype Identification

The 50 most expressed markers for each airway cell subtype were extracted from the “Lung Atlas: Epithelial Cells” dataset from the “Teichmann Lung and Asthma Atlases—available from UCSC Cell Browser (https://cells.ucsc.edu) [2]. The Log_2_FC values obtained from our RNA-seq dataset were then matched to these genes when present.

#### 2.9.3. Patterns of Gene Expression and Signaling Pathways Using NetworkAnalyst

NetworkAnalyst (https://www.networkanalyst.ca, [55]) was used to identify specific patterns of gene expression and signaling pathways (KEGG—Kyoto Encyclopedia of Genes and Genomes—and Reactome analysis), biological processes, molecular functions. and cellular compartments (PANTHER analysis) that were significantly regulated by the differentiation media. Organism (*H. sapiens*) and ID type (ensemble gene ID) were specified and lists of upregulated and downregulated genes were uploaded along with the corresponding Log_2_FC. The list enrichment network of the assorted visual analytics was then selected and enrichment analysis performed using the KEGG, Reactome, PANTHER BP (Biological Process), MF (Molecular Function), and CC (Cellular Component) databases. Lists were extracted and resulting Gene counts and Log(*p*-value) plotted using GraphPad Prism8.

#### 2.9.4. Statistical Analysis of Other Data

All other analyses were performed using GraphPad Prism8 (GraphPad, San Diego, CA, USA). Where applicable, results are shown as mean ± sem. Parametric and non-parametric data distributions were assessed with the D’Agostino and Pearson normality test. Multiple group and two-group comparisons were performed using appropriate statistical tests for specific data sets (see details in individual figure legends).

## 3. Results

### 3.1. UNC and SC Media Induce Distinct Epithelial Phenotypes in CF and NCF Epithelia

The effect of growth media on epithelial integrity was first investigated by measuring the TEER of CF and NCF monolayers. SC-grown epithelia produced a significantly “leakier” phenotype compared to UNC-grown cells (Figure 2A, CF SC, 294 ± 21 Ω·cm^2^ vs. UNC, 801 ± 45 Ω·cm^2^, *p* < 0.0001; NCF SC, 156 ± 5 Ω·cm^2^ vs. UNC, 629 ± 42 Ω·cm^2^, *p* < 0.0001). Interestingly, CF hAECs had a higher TEER than NCF cells for both differentiation media (Figure 2A). Initial phenotypic analysis showed no apparent difference in either acidic or neutral mucin staining between UNC- and SC-grown epithelial cells (Figure 2B). SC-grown cultures had a significantly increased epithelial thickness for both CF and NCF cultures compared to UNC-grown cells (Figure 2C, CF SC, 20 ± 2 μM vs. UNC, 8.1 ± 0.7 μM, *p* < 0.0001; NCF SC, 25 ± 2 μM vs. UNC, 8.2 ± 0.7 μM, *p* < 0.0001). Further phenotypic characterization was then performed by immunofluorescence (Figure 2 D–I). Both SC-grown CF and NCF epithelia showed a greater cell density as assessed by the number of nuclei (Figure 2F), as well as a marked increase in the number of ciliated cells (α-tubulin, Figure 2D,G). In contrast, the number of mucin-producing cells (MUC5AC positive) was not affected by the differentiation medium (Figure 2D,H). Interestingly, growth medium induced a significant increase in the amount of actin filaments in NCF cells only (Figure 2I), and also affected the intracellular distribution of F-actin in both CF and NCF cells: while F-actin was localized on the same z-plan as α-tubulin in UNC-grown epithelia, it was located just below α-tubulin staining in SC-grown cultures (Figure 2D,E).

### 3.2. CF Cells are More Susceptible to Changes in Growth Media than NCF Cells

**Figure 3:**
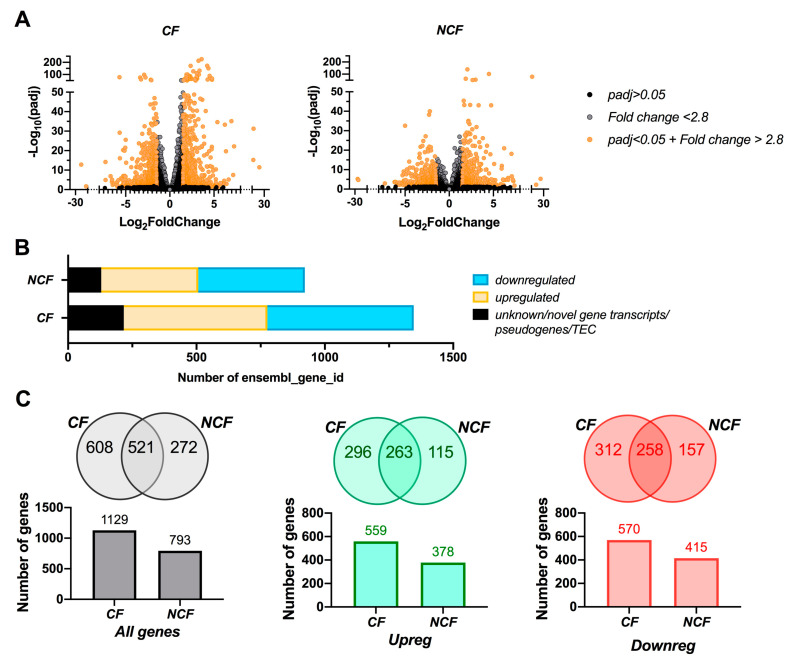
Transcriptomic analysis of CF and NCF hAECs grown in UNC vs. SC media. (**A**) Volcano plots displaying differentially expressed transcripts between UNC- and SC-grown CF (left panel) and NCF (right panel) cells. The Log_10_(adjusted *p*-values) were plotted against the Log_2_FC in gene expression. Genes upregulated or downregulated (*n* = 916) by 2.8-fold or more (Log_2_FC > 1.5) and with a *p*-adj < 0.05 are depicted as orange dots. All other genes that were not found to be differentially expressed are depicted as grey and black dots. (**B**) Number of transcripts that were up- (yellow) or down-regulated (blue) in UNC compared to SC-grown CF and NCF cells as well as unknown transcripts and pseudogenes (black). (**C**) Venn diagrams and bar graphs representing the total number of genes (grey, left panels), upregulated (green, middle panels) and downregulated (red, right panels) shared between CF and NCF hAECs differentiated in UNC compared to SC media.

To further investigate the impact of differentiation medium on hAECs, an in-depth genotypic analysis was performed by RNA-Seq from UNC- and SC-grown CF and NCF cultures. Transcriptome analysis found 20,330 and 19,052 transcripts in CF and NCF epithelia, respectively. Cut-offs of 1.5 and 0.05, for Log_2_FC (equivalent to a 2.8-fold change) and adjusted *p*-value (*p*-adj), respectively, were used to determine the significantly up- or down-regulated transcripts (Figure 3A). Further analysis revealed that 1346 genes were significantly regulated in CF cells compared to 922 in NCF cells (Figure 3B). Of these, 217 and 129 were novel gene transcripts or pseudogenes in CF and NCF cells, respectively. In CF cells, 559 genes were upregulated and 570 genes were downregulated in UNC medium, when compared to SC medium (Figure 3B). In NCF cells, 378 genes were upregulated and 415 genes were downregulated in UNC medium, when compared to SC medium (Figure 3B). Figure 3C represents the number of shared genes between CF and NCF cells that were significantly regulated in the two differentiation media. Most genes that were differentially regulated in NCF cells were found in CF-regulated genes pool (65% total genes, left panel; 70% upregulated genes, middle panel; 62% downregulated genes, right panel), whereas the opposite was not true, showing that differentiation media affected the transcriptome of CF cells more than NCF cells. As focusing on CF cells allows for a more specific characterization of the cellular and molecular changes induced by the differentiation media, the remainder of this paper will focus on the impact of the two media on CF cells only.

### 3.3. Growth Conditions Dictate Airway Epithelial Cell Identity

**Figure 4:**
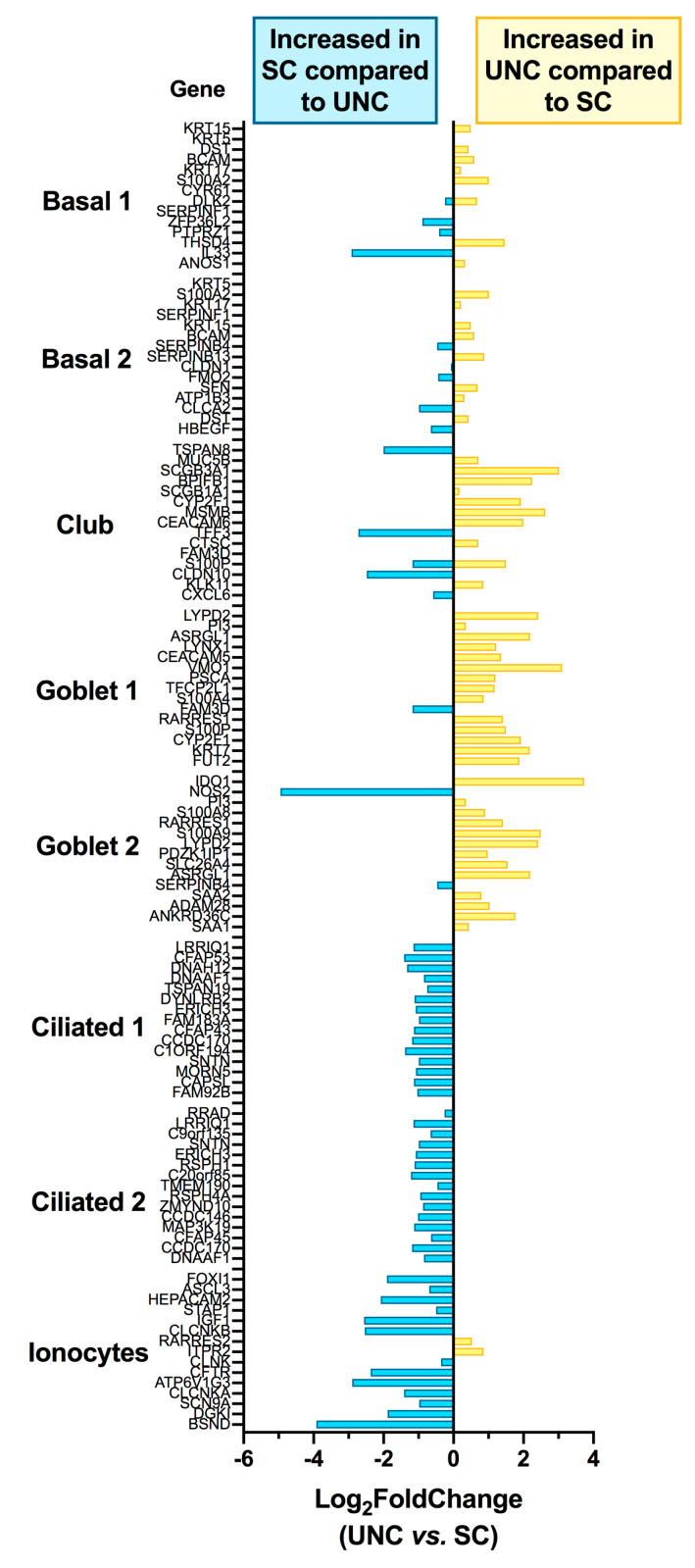
Transcriptomic analysis of CF hAECs grown in UNC vs. SC media shows differentiation into airway epithelial cell subtypes depends on media. Most expressed genes per airway epithelial cell type were extracted from the dataset “Lung Atlas: Epithelial Cells” from the “Teichmann Lung and Asthma Atlases—available from UCSC Cell Browser (https://cells.ucsc.edu). The Log_2_FC, for the 15 most expressed genes for each cell type, were then extracted from our transcriptomic study and plotted on the *x*-axis.

The RNA-seq dataset was next used to investigate the impact of the two media on epithelial cell subtype markers of CF cultures. The 50 most expressed markers for each airway cell subtype were extracted from the “Lung Atlas: Epithelial Cells” dataset from the “Teichmann Lung and Asthma Atlases—available from UCSC Cell Browser (https://cells.ucsc.edu) to which the Log_2_FC values obtained from our RNA-Seq dataset were matched. The 15 most expressed markers for each cell subtype are presented in Figure 4.

On average, for each set of 50 genes, the results show that basal cell markers (Log_2_FC Basal 1 = −0.1015 ± 0.1434; Basal 2, −0.04046 ± 0.1190) were not affected by differentiation media but that genes associated with club, goblet 1, and goblet 2 subtypes were significantly increased in UNC-grown epithelial cells compared to SC by 1.6 fold (Log_2_FC = 0.6596 ± 0.2134), 2.1 fold (Log_2_FC = 1.060 ± 0.1698), and 1.9 fold (Log_2_FC = 0.9173 ± 0.2001), respectively. Conversely, markers of ciliated 1 and 2 and ionocytes were increased in SC-grown cells compared to UNC-grown cultures by 2.2 fold (Log_2_FC = −1.106 ± 0.0387), 2 fold (Log_2_FC = −0.9802 ± 0.0549) and 2.1 fold (Log_2_FC = −1.070 ± 0.2491).

### 3.4. Pathway Analysis Reveals a Differentiation Medium Dependent Shift in Expression of Genes Involved in Ion and Fluid Transport Homeostasis

**Figure 5:**
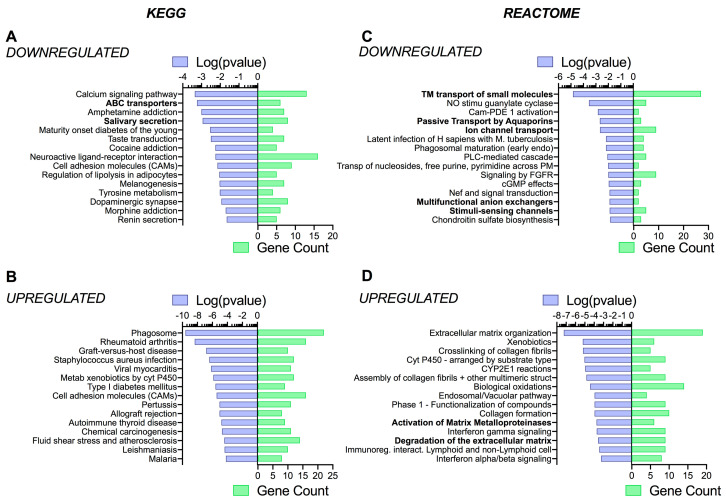
KEGG (Kyoto Encyclopedia of Genes and Genomes) (**A**,**B**), and Reactome (**C**,**D**) enrichment analysis. Top 15 pathways associated with downregulated (**A**,**C**) and upregulated (**B**,**D**) mRNAs in CF hAECs grow in UNC compared to SC media. Pathways identified are represented on the *y*-axis. The left *x*-axes correspond to the log of the *p*-value (purple) and the right *x*-axes correspond to the number of significant genes found in a given pathway (green).

NetworkAnalyst [55] was then used to assist in understanding the specific patterns of changes in gene expression, as well as determine whether some signaling pathways (KEGG—Kyoto Encyclopedia of Genes and Genomes—and Reactome analysis), biological processes, molecular functions, and cellular components (PANTHER analysis) were dependent on the differentiation medium. Although there were slight differences in *p*-values and gene counts (related to the total number of genes differentially regulated, see paragraph 3.2) between CF and NCF cells, a similar pattern of pathways and functions specific to epithelial cells was observed. Therefore, the main differences observed in CF cells are reported here (NCF cell pathway analysis data are shown in Figures S1 and S2). Indeed, using the KEGG and Reactome databases, we found that ‘ABC transporters’, ‘salivary secretion’, ‘transmembrane transport of small molecules’, ‘passive transport by aquaporins’, ‘ion channel transport’, ‘multifunctional anion exchangers’, and ‘stimuli-sensing channels’, respectively, were all downregulated in UNC medium compared to SC medium (Figure 5A,C). KEGG and Reactome pathways upregulated in UNC medium were those related to ‘infection’ (KEGG) and ‘protease activity’ (Reactome) (Figure 5B,D). 

**Figure 6:**
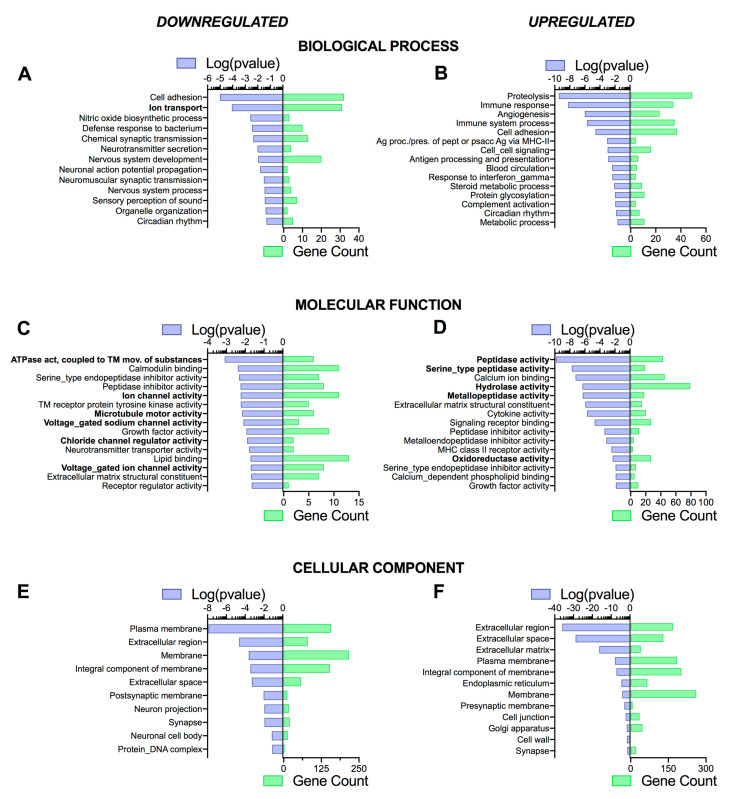
PANTHER enrichment pathway analysis of downregulated (**A**,**C**,**E**) and upregulated (**B**,**D**,**F**) genes in UNC- compared to SC-grown CF hAECs. Top 10–15 items of biological process (**A**,**B**), molecular function (**C**,**D**), and cellular component (**E**,**F**) pathways using PANTHER database. Pathways identified are represented on the *y*-axis. The left *x*-axes correspond to the log of the *p*-value (purple) and the right *x*-axes correspond to the number of significant genes found in a given pathway (green).

The use of PANTHER databases showed an upregulation in ‘ion transport’, ‘ATPase activity coupled to transmembrane movement of substances’, ‘ion channel activity’, ‘voltage-gated sodium channel activity’, ‘chloride channel regulator activity’, and ‘voltage-gated ion channel activity’ when CF cells were grown in SC medium compared to UNC (Figure 6A,C). Interestingly, this analysis also showed a decrease in ‘microtubule motor activity’ in the UNC condition, suggesting that SC medium could upregulate cilia function (Figure 6C). PANTHER analysis also reported an increase in ‘proteolysis’, ‘immune response’, and various enzymes activities (peptidases, hydrolase, oxidoreductase) in cells grown in UNC medium compared to SC (Figure 6B,D). Performing the analysis using the cellular component PANTHER database did not reveal any major differences between CF cells grown in UNC and SC media (Figure 6E,F). Taken together, the pathway analysis of the transcriptomic data suggests that differentiation media specifically targeted genes involved in ion and fluid transport. As this is of specific interest and very relevant to CF research, the rest of the paper focuses on this aspect.

### 3.5. UNC-Grown CF Cells Express Higher Levels of ATP12A

**Figure 7:**
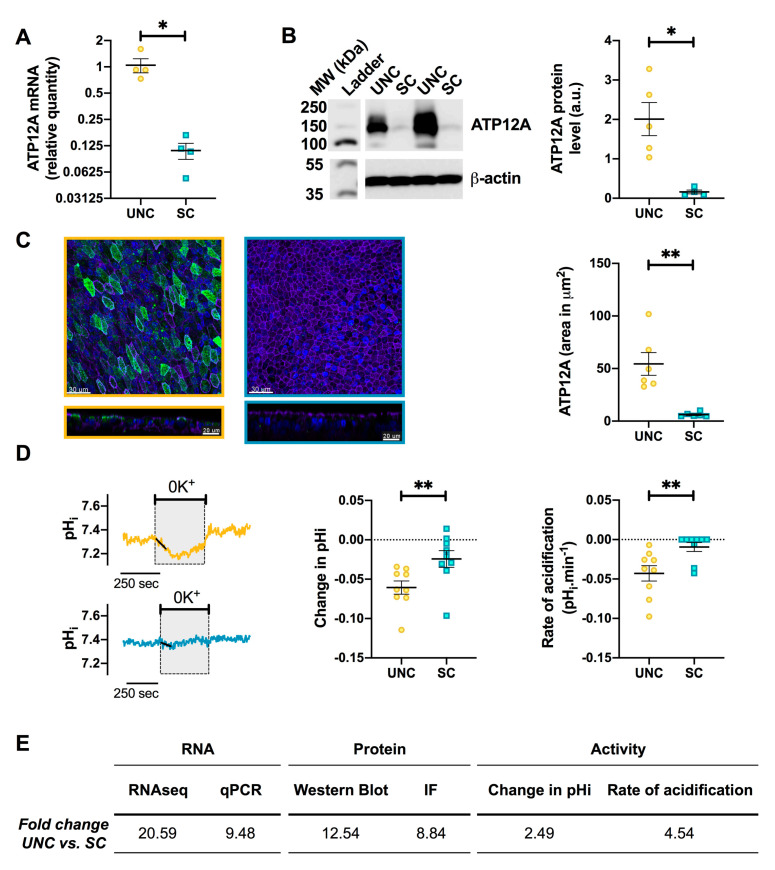
UNC-grown CF cells show higher ATP12A expression and activity. (**A**) ATP12A mRNA levels were measured in UNC- and SC-grown CF hAECs by RT-qPCR and reported as relative quantity (RQ) using the 2^−ΔΔCt^ method. (**B**) Left panel shows a representative Western blot of ATP12A protein levels in UNC- and SC-grown CF hAECs. Right panel represents the quantification by densitometry of the ATP12A bands relative to the loading control, β-actin. (**C**) Left upper panels show representative XY immunofluorescence (IF) images of UNC- (yellow) and SC-grown (blue) CF hAECs with ATP12A shown in green, nuclei in blue, and F-actin in purple. Lower panels show the XZ plans of the upper images. Right panel shows the quantification of ATP12A in the IF images. (**D**) Measurement of ATP12A activity by intracellular pH measurements. Left panels show pH_i_ recordings before, during, and after exposure to apical K^+^-free solution (0K^+^) of CF hAECs grown in UNC (yellow, upper trace) and SC (blue, lower trace). ATP12A activity was measured as the 0K^+^-induced change in pH_i_ (middle panel) and rate of acidification (right panel). (**E**) Summary of fold changes in ATP12A mRNA, protein, and activity levels in UNC-grown CF hAECs compared to SC-grown CF hAECs. For all graphs, UNC-grown cells are in yellow, SC-grown cells in blue, black lines and error bars represent mean ± sem, Mann-Whitney test; * *p* < 0.05, ** *p* < 0.01.

The most differentially regulated gene, involved in ion transport, was ATP12A in both CF and NCF cells (CF: Log_2_FC = 4.364; NCF: Log_2_FC = 4.492). ATP12A is a H^+^/K^+^ ATPase that has previously been shown to contribute to a decreased ASL pH in CF [56] and therefore is a potential therapeutic target for normalizing ASL pH in CF airways [57]. In order to confirm the RNA-Seq result, RT-qPCR was performed, and this showed a significant downregulation of ATP12A mRNA in SC-grown CF hAECs (Figure 7A). This result was further strengthened by the demonstration of significantly lower protein expression by Western blot (Figure 7B), as well as immunofluorescence in SC-grown CF hAECs (Figure 7C). 

To evaluate whether lower mRNA and protein expression translated into lower activity, ATP12A functionality was assessed by intracellular pH (pH_i_) experiments [57]. In UNC-grown CF epithelia, exposure of the apical surface to a K^+^-free modified Krebs solution (0K^+^) induced an intracellular acidification of −0.06 ± 0.01 at a rate of −0.04 ± 0.01 pH·min^−1^, due to the inhibition of H^+^ secretion by ATP12A, which accumulated in the cytosol (Figure 7D). On the other hand, exposure of SC-grown CF hAECs to 0K^+^ produced a significantly smaller acidification compared to UNC-grown CF epithelia (SC ΔpH_i_ = −0.02 ± 0.01, *p* = 0.006; rate of acidification: −0.01 ± 0.01, *p* = 0.004). Fold changes in mRNA, protein, and levels of activity are reported in Figure 7E and show that a 10 to 20 fold change in mRNA levels induced a 2.4- to 4.5-fold increase in H^+^ transport (Figure 7E). Taken together, these results show that there exists a significant difference in ATP12A mRNA, protein, and functional activity between UNC- and SC-grown cells that could impact airways pH homeostasis, which is known to be deregulated in CF airways [42,43,56,57,58].

### 3.6. CFTR Expression and Function are Altered by Differentiation Media in CF Cells

**Figure 8:**
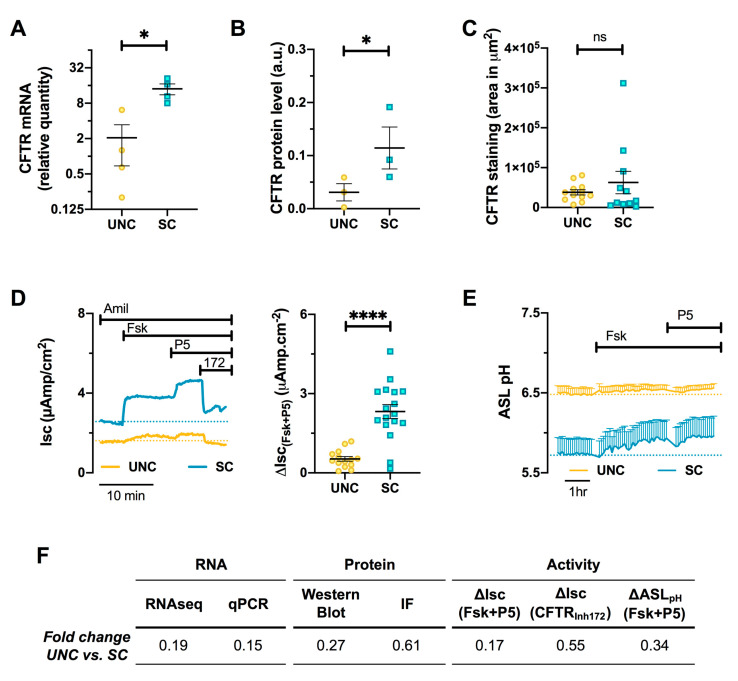
UNC-grown CF cells show lower CFTR expression and activity. (**A**) CFTR mRNA levels were measured in UNC- and SC-grown CF hAECs by RT-qPCR and reported as RQ using the 2^−ΔΔCt^ method. (**B**) CFTR protein levels were measured by Western blot. Quantification was performed by densitometry of the CFTR bands relative to the loading control, β-actin. (**C**) CFTR protein levels were measured by quantification of CFTR positive area in the immunofluorescence; ns: non significant. (**D**) Measurement of CFTR activity in Ussing chamber experiments. Left panel shows representative short-circuit currents (Isc) recordings of CF hAECs grown in UNC (yellow trace) and SC (blue trace) media. CFTR activity was measured as the change in Isc (ΔIsc) induced by forskolin (Fsk, 10 μM, bilateral), potentiator P5 (2 μM, basolateral), and CFTR_Inh172_ (20 μM, apical). Right panel shows the change in Isc induced by Fsk and P5. (**E**) CFTR activity was also measured in recordings of airway surface liquid (ASL) pH of CF hAECs grown in UNC (yellow trace) and SC (blue trace) media. (**F**) Summary of fold changes in CFTR mRNA, protein and activity levels in UNC-grown CF hAECs compared to SC-grown CF hAECs. For all graphs, UNC-grown cells are in yellow, SC-grown cells in blue, black lines and error bars represent mean ± sem, all panels except E-right: Mann-Whitney test, panel E-right: 2-Way ANOVA with Sidak’s multiple comparisons test; * *p* < 0.05, **** *p* < 0.0001.

RNA-Seq data also showed a significant increase in F508del-CFTR mRNA levels when CF hAECs were differentiated in SC compared to UNC media (UNC vs. SC Log_2_FC = −2.37, *p*-adj = 3.17.10^−9^) that was confirmed by RT-qPCR (Figure 8A). This SC-induced increase in mRNA also resulted in higher (although weak) protein expression, as measured by Western blot (Figure 8B). 

Although CFTR mRNA and protein levels were increased by SC medium compared to UNC, there was no difference in immunofluorescence staining of the CFTR channel (Figure 8C). To investigate whether this increase in CFTR expression had any functional impact on the epithelium, the Cl^−^ and HCO_3_^−^ secretory function of delF508-CFTR was evaluated by measuring transepithelial transport (Isc) and ASL pH, respectively. In CF hAECs Cl^−^, secretion was measured by the Isc response to forskolin (Fsk) and the CFTR potentiator, P5, as well as the total Isc inhibited by the specific CFTR inhibitor, CFTR_Inh172_ (172), after first inhibiting sodium transport by ENaC with amiloride (Figure 8D, left panel). Figure 8D shows that growing cells in SC medium induced significant increases in Fsk, P5, and CFTR_Inh172_-induced changes in short-circuit currents (ΔIsc) compared to UNC-grown cells, consistent with more F508del-CFTR at the cell surface. The capacity of CFTR to secrete bicarbonate was also investigated by ASL pH experiments. Figure 8E shows the mean change in ASL pH of CF hAECS grown in UNC (yellow trace) or SC (blue trace) before, and after, the addition of Fsk and P5. Surprisingly, resting ASL pH was lower in SC- than UNC-grown cultures, and although it appeared that Fsk and P5 increased ASL pH of SC-grown cells (Figure 8E), this did not reach significance. A summary of the fold changes in mRNA, protein, and activity levels for UNC vs. SC media is shown in Figure 8F. Taken together, these results demonstrate that although SC differentiation media affected F508del-CFTR expression, it had a minor, but measurable, impact on its function.

### 3.7. Differentiation Media Modulate CF Cells Response to CFTR Modulators

**Figure 9:**
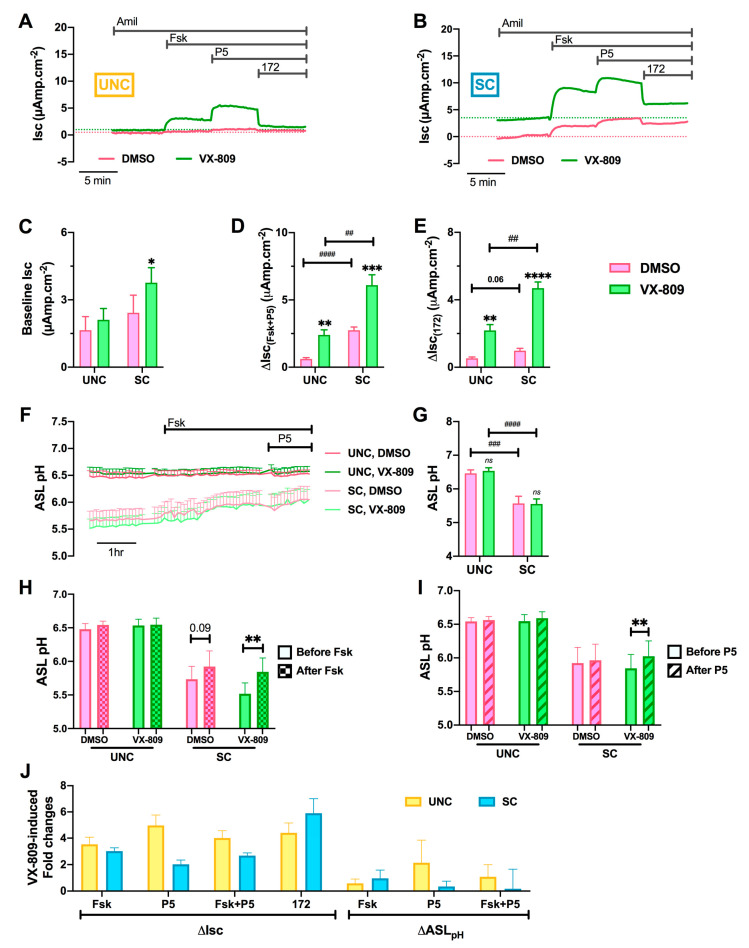
Differentiation media affects the functional response to CFTR modulators. (**A**,**B**) Representative recordings of CFTR activity, in Ussing chamber experiments, of CF hAECs grown in UNC (**A**) or SC (**B**) media in the presence (green trace) or absence (pink trace) of the CFTR corrector VX-809 (3 μM, 48 hr, basolateral). The experiments were performed in the presence of 10 μM apical amiloride (Amil) and changes in Isc were recorded following addition of forskolin (Fsk, 10 μM, bilateral), P5 (2 μM, basolateral), and CFTR_Inh172_ (172, 20 μM, apical). (**C**–**E**) Summary data showing CFTR activity measured as the change in Isc (ΔIsc) induced by forskolin (Fsk, 10 μM, bilateral, (**C**)), potentiator P5 (2 μM, basolateral, (**D**)), and CFTR_Inh172_ (172, 20 μM, apical, (**E**)). (**F**) Measurements of ASL pH in CF hAECs grown in UNC (dark-pink and dark-green traces) or SC (light-pink and light-green traces) media in the presence (green traces) or absence (pink traces) of the CFTR corrector VX-809 (3 μM, 48 hr, basolateral). (**G**) Summary data of the effect of VX-809 on resting ASL pH in UNC- and SC-grown CF hAECs. (**H**,**I**) Summary data of the effect of VX-809 on changes in ASL pH induced by Fsk (**H**) and P5 (**I**). (**J**) Summary diagram of the VX-809-induced fold changes, calculated as [response in the presence of VX-809/response in the presence of DMSO], for CFTR activity, measured in Ussing chamber and ASL pH experiments, for UNC- (yellow) vs. SC-grown (blue) CF hAECs. For graphs (**A**–**J**), DMSO-treated cells are in pink and VX-809-treated cells are in green. Graphs (**C**–**J**) are mean ± sem. Graph (**J**): UNC-grown cells are in yellow, SC-grown cells in blue. Panels D and E: # is for UNC vs. SC comparison, * is for VX-809 vs. DMSO comparison; * *p* < 0.05, ** *p* < 0.01, ***p < 0.001, **** *p* < 0.0001, ## *p* < 0.01, ### *p* < 0.001, #### *p* < 0.0001.

Because CFTR mRNA and protein levels were differentially affected by the two differentiation media, the response of the cells to CFTR modulators was then investigated. For these experiments, CF hAECs grown in UNC or SC media were pre-treated with the corrector VX-809 (3 μM) or vehicle (DMSO 0.1 % *v*/*v*) for 48 h and then CFTR-dependent ion transport evaluated (Figure 9A,B), using the same protocol as described in Figure 8. VX-809 increased resting Isc in SC-grown cultures only (Figure 9B,C). For both UNC- and SC-grown CF epithelia, VX-809 pre-treatment significantly increased the response to Fsk and P5 (Figure 9D), and CFTR_Inh172_ (Figure 9E), although the absolute changes in Isc were larger in SC- than UNC-grown CF epithelia (Figure 9D,E).

There have been reports of CFTR modulators impacting the chloride and bicarbonate conductance of CFTR differently [59,60], and, therefore, the effect of the two differentiation media on bicarbonate secretion into the ASL was investigated in VX-809-corrected CF hAECs. ASL pH was recorded in CF hAECs grown in UNC (dark traces) or SC (light traces) in the presence (green traces) or absence (pink traces) of VX-809 before and after addition of Fsk. As previously observed in untreated cells (Figure 8), SC-grown CF cultures pre-treated with VX-809 showed a more acidic resting ASL pH than UNC-grown cells (Figure 9G). There was no effect of VX-809 treatment on resting ASL pH of hAECs grown in either media. Furthermore, pre-treatment with VX-809 did not affect cell responses to Fsk in either UNC or SC cultures (Figure 9H). Although small, VX-809 induced a significant increase in P5-stimulated ASL pH in SC-grown CF hAECs only (Figure 9I). Finally, the VX-809-induced fold changes in functional F508del-CFTR activity, as measured by Isc as well as by ASL pH, was calculated. Interestingly, although not significant, P5 induced a bigger fold change in Isc in UNC differentiated CF hAECs compared to SC-grown cultures (FC ΔIsc(P5) UNC = 4.97 ± 0.79, SC = 2.02 ± 0.32, *p* = 0.085; FC ΔASL pH(P5) UNC = 2.14 ± 1.71, SC = 0.35 ± 0.41, *p* = 0.539). Taken together, these results strongly suggest that both differentiation media affected the Cl^−^ and HCO_3_^−^ conductance of CFTR differently, particularly after treatment with VX-809, which is important in the context of CFTR modulator therapies.

## 4. Discussion

Fully differentiated cultures of primary human airway cells grown at ALI are now considered the gold standard for in vitro studies on airway biology under normal or disease conditions [25,26]. Specifically in CF research, the use of such differentiated ALI cultures has been instrumental in the development of CFTR-directed therapies for people with CF [38,39]. However, there are important limitations of this approach, which need to be taken into consideration. Growth factors are well known to have profound effects on cell differentiation [61,62]. Therefore, it is not surprising that the choice of growth factors plays a fundamental role when optimizing any in vitro model [63]. However, because we studied the effects of two commonly used culture media that had been optimized for the growth and differentiation of large airway epithelial cells, we were very surprised that they led to such profound differences in cellular differentiation and functional responses. Our results show that epithelial architecture as well as cell density were strongly impacted by the choice of medium. In addition, RNA-seq revealed a profound shift in airway cell lineage, with the UNC medium promoting differentiation into Club and Goblet cells whilst the SC medium stimulated a very marked increase in multiciliated cells, as well as ionocytes. Of note, the different databases, used for the pathway analysis of the RNA-seq datasets, identified different regulated pathways. It is known that there exists some heterogeneity between databases [64]. However, the results returned from these analyses were globally consistent with a regulation of fluid and ion transport systems (Figure 5 and Figure 6).

Although a reduced TEER was observed in SC- compared to UNC-grown epithelia, this was not because the cells formed a leakier epithelium containing fewer tight junctions, as SC cultures demonstrated robust tight junctional protein expression with an increased cell density. Tight junctions help the development of cell polarity, provide for cell–cell adhesion, act as a permeability barrier in well-differentiated epithelia, and also regulate the paracellular flux of ions by creating charge-selective pores, whose selectivity is mainly determined by claudin expression [65,66]. Our RNA-seq data showed differences in claudin expression in the two different CF models (Table S3). Claudins can form ion-selective pores but can also have a sealing function. Therefore, it is possible that the reduced TEER measured in SC epithelia is due to a combination of reduced expression of sealing claudins, combined with an increase in electrogenic ion transport in SC cultures. In a wider context, the differential expression of claudins is very interesting, since tight junction manipulation has been suggested as a possible therapy in CF [67].

One of the most controversial areas in CF research is whether the pH of the ASL is the same, or is more acidic, in CF compared to non-CF, due to reduced HCO_3_^−^ secretion via dysfunctional CFTR [68]. A more acidic pH in CF has been observed both in vitro [69] as well as in vivo, in neonates at least [70], and has been linked to causing excessive Na^+^ absorption [71], mucus dehydration [44], and reduced bacterial killing [69,72,73] of CF cultures. Indeed, many researchers consider that the more acid ASL pH in CF actually initiates inflammation and host defense abnormalities, leading to early bacterial colonization of the airways [56]. However, these results have not been confirmed by other studies, both in vivo and in vitro [74,75]. Even though these in vitro studies employed different growth conditions, whether this explains the discrepant results is not entirely clear. For example, Schultz et al. used the UNC growth media, i.e., the same in vitro growth conditions as the current study, and their ASL pH results agree with our previously published results, which again used UNC media, and which also did not show significant differences in the steady-state pH of the ASL at rest between non-CF and CF cultures although the ability of cAMP agonists to increase ASL pH was completely absent in CF cultures [53]. In addition to CFTR, the activity of the proton ATPase, ATP12A has also been shown to play an important role in ASL pH homeostasis, both in vitro [76] and in vivo [56]. In the current study, ATP12A was one of the most up-regulated genes in CF UNC cultures compared to SC-grown cultures, which was confirmed by a combination of RT-qPCR, protein expression, and functional studies. Previously, it has been shown that ATP12A was mainly expressed in goblet cells, both in vivo and in vitro [77]. This clearly explains the increased expression of ATP12A in the UNC epithelia, which favored differentiation into goblet cells (Figure 4), making this model particularly useful for studying this protein. It was therefore expected that UNC-grown cells would show a more acid ASL than SC, but the opposite was the case. Despite SC-grown cells showing little ATP12A activity (and more residual F508del-CFTR activity), steady-state ASL pH was significantly lower in SC-grown CF cultures (Figure 8E, left panel). The reason for this is not currently known, but it suggests that other channels/transporters may also impact ASL pH homeostasis. For example, Garnett et al. showed that primary airway cells have robust lactate transport through monocarboxylate transporters (MCT), which serves to acidify ASL pH in vitro [78]. However, our RNA-seq data showed that these transporters were either unchanged or even upregulated in UNC-grown cells, thus excluding this hypothesis (Table S4). Pendrin (SLC26A4) has previously been shown to regulate ASL pH in vitro, in both airway cell lines [79] and primary epithelial cells [80]. According to our RNA-seq data, pendrin expression was significantly upregulated in UNC CF epithelia (UNC vs. SC Log_2_FC = 1.54, *p*-adj = 7.36.10^−5^), which therefore provides a potential explanation for the higher ASL pH in UNC-grown cells, despite these cells having active secretion of protons. The higher resting ASL pH in UNC-grown cells could also be due to differences in paracellular flux of anions, particularly HCO_3_^−^, across the tight junctions. Recently, Thornell et al. studied the HCO_3_^−^ permeability of the paracellular pathway in CF and NCF bronchial epithelia. They showed that at a steady state, HCO_3_^−^ flux was weakly secretory and could be modulated by cytokines, suggesting that paracellular HCO_3_^−^ transport could offset changes in ASL pH induced by transcellular movement of ions [81]. Moreover, a significant HCO_3_^−^ permeability of the tight junctions in UNC-grown cells is consistent with previous results by Coakley et al., which also used cells grown in UNC media [76]. In this study, they showed that cultures exposed to an acute acid load to the luminal surface, recovered their ASL pH relatively quickly, which they suggested was due to a high constitutive HCO_3_^−^ flux across the tight junctions [76]. However, the fact that ASL pH of SC-grown cells was significantly different to the media pH (~7.4) strongly implies that SC-grown cells do actively acidify luminal surface fluid. Since ATP12A was not functional in these cells (Figure 7D), other candidate apical H^+^ transporters (or base-importers) must be responsible. Potential candidates include H^+^ channels and sodium-hydrogen exchangers (NHEs), both of which have been shown to be active in airway cells [82] and be important for a proper mucociliary differentiation [83]. The RNA-seq data showed that UNC-grown epithelia overexpressed two NHE isoforms: NHE2 (SLC9A2) and NHE4 (SLC9A4), whereas differentiation media had little effect on H^+^ channels’ expression (Table S5). These NHEs are known to be present in gastric mucus cells and are responsible for regulating intracellular pH and cell volume, respectively [84], but have not been shown to be functionally present in airway cells, so further work investigating NHE activity would be informative. These results provide a clear example that it is not possible to simply compare results obtained by different cell culture protocols without first establishing the functional phenotype of the cells in question.

Our study also showed that the two culture media had distinct effects on the expression and trafficking of F508del-CFTR (Figure 8); indeed CF epithelia grown in SC medium showed a significantly larger Isc response to cAMP agonists and CFTR potentiator. Treating these CF cells with the corrector, VX-809, further emphasized these differences. The fact that the activity and correction of F508del-CFTR was different between SC- and UNC-grown epithelia could be explained by enhanced trafficking and/or retention of F508del-CFTR at the cell surface, due to a better developed F-actin web in UNC-grown cells, as detected by the IF images in this study (Figure 2), and as previously described [85]. Another potential explanation for the higher basal activity of F508del-CFTR in SC-grown cells is that the immature protein trafficked to the apical membrane by utilizing the non-conventional secretory pathway [86]. This idea is supported by the RNA-seq results, which showed that MVB12B was more expressed in SC-grown epithelia (UNC vs. SC Log_2_FC = −1.17, *p*-adj = 8.04 × 10^−4^). MVB12B has been shown to physically interact with immature band B F508del-CFTR, and is the most relevant gene involved in unconventional CFTR secretion [87]. This is also consistent with our WB results, which mainly detected CFTR band B. Although we found higher expression of F508del-CFTR mRNA (in both RNA-seq and qPCR data) in SC-grown cells, there was only a minor increase in protein expression (Figure 8). This led us to consider whether the degradation of the mutant CFTR protein could account for these results. In the literature, the transcriptional and post-transcriptional CFTR regulation pathway is well described [88]. Misfolded F508del-CFTR is sequestered in the ER and then degraded through the ER-associated degradation (ERAD) pathway, which involves many co-chaperones [89]. Although our RNA-seq data revealed that a number of proteins that interact with CFTR were differentially regulated in UNC- and SC-grown epithelia (Table S6), it is unclear whether enhanced protein degradation via ERAD could account for our results.

Finally, one interesting observation from short-circuit current measurements was the finding that the CFTR-specific inhibitor (CFTR_Inh172_) was unable to block all the Fsk+P5-induced current (Figure 8) in CF cells, which suggests the activation of another Cl^−^ channel or electrogenic transporter. A possible candidate is SLC26A9, which interacts with CFTR and enhances Fsk-induced currents [90], and was found to be upregulated in SC epithelia (UNC vs. SC Log_2_FC = −1.87, *p*-adj = 0.04). However, current evidence suggests that SLC26A9 protein expression is reduced in CF cells because of its interaction with F508del-CFTR at the level of the ER, and thus it also fails to traffic to the cells’ surface [91,92]. Nonetheless, if F508del-CFTR traffics to the cell surface via the unconventional pathway (see above), then this could provide an explanation for the presence of SLC26A9 as well. Overall, these findings have important implications when in vitro cell cultures are used for drug discovery [93], particularly in the case of CFTR modulators [94] and more recently for personalized therapy, especially in the case of very rare CFTR mutations [95], in which choosing the right cell growth method could lead to findings that may not be replicated by a different growth protocol.

## 5. Conclusions

Significant progress has recently been made in the characterization of the different cell types that compose the conducting airways through advances in single cell RNA-seq [96] and a better understanding of cell differentiation pathways [97,98]. Therefore, it is now possible to know which genes drive the differentiation of different cell types, and how to control their expression in order to better regulate cell niche choice and differentiation. It is therefore important to be aware of this when choosing a growth medium for a specific research project, especially if one employs a commercial medium in which some of the key components, such as growth factors, will not be known and that might impact the proportion of specific cell types such as ciliated cells or ionocytes. This study provides a good illustration where two commonly used growth media differentially impacted on epithelia gene expression, differentiation, morphology, and the activity of key ion channels. Growth conditions also affected the response to CFTR modulators, which highlights the need for better awareness, and potentially the need for standardization of this parameter in airways research.

## Figures and Tables

**Table 1:** Gene, primer orientation, primer sequence (5′ to 3′), and predicted product lengths for primers used in RT-qPCR assays.

Target Gene	Primer	Sequence	Product Length
*18S rRNA*	Forward	5′-CTCTAGATAACCTCGGGCCG-3′	209
	Reverse	5′-GTCGGGAGTGGGTAATTTGC-3′	
*ATP12A*	Forward	5′-GGGGCACACTTGTTCATCTTCTGA-3′	128
	Reverse	5′-GCAAAACATCAGTGAGCATCCTG-3′	
*CFTR*	Forward	5′-AGGAGGCAGTCTGTCCTGAA-3′	237
	Reverse	5′-CACTGCTGGTATGCTCTCCA-3′	

**Table 2:** Antibodies used in Western-blotting (WB) and immunostaining (Immunofluorescence, IF).

Protein Target	Manufacturer (Reference)	Species Raised in	Dilution/Concentration (WB/IF)
***Primary Antibodies***			
β-actin	Sigma-Aldrich (A5441)	Mouse	1:5000 (WB)
CFTR (Cystic Fibrosis transmembrane conductance regulator	CFF Therapeutics (596)	Mouse	1:3000 (WB)
			1:250 (IF)
ATP12A	Sigma-Aldrich (HPA039526)	Rabbit	1:1000 (WB)
			1:400 (IF)
MUC5AC	Abcam (ab3649, Cambridge, UK)	Mouse	1:67 (IF)
Acetyl-α-Tubulin (Lys40) (D20G3)	Cell Signaling Technology (5335, London, UK)	Rabbit	1:800 (IF)
Phalloidin (Alexa Fluor 647)	Thermo Fisher Scientific (A30107)	NA	1.25 µg/mL
DAPI (4′,6-diamidino-2-phenylindole)	Sigma-Aldrich (D9542)	NA	1:1000
***Secondary Antibodies***			
Anti-mouse Immunoglobulin (Ig)G, HorseRadish Peroxidase (HRP)-linked	Cell Signaling Technology (7076)	Horse	1:5000 (WB)
Anti-rabbit IgG, HRP-linked	Cell Signaling Technology (7074)	Goat	1:5000 (WB)
Precision Protein™ StrepTactin-HRP Conjugate	Bio-Rad (161038)	NA	1:5000 (WB)
Anti-Rabbit IgG (Alexa Fluor 488)	Thermo Fisher Scientific (A11034)	Goat	5 µg/mL (IF)
Anti-Mouse IgG (Alexa Fluor 594)	Thermo Fisher Scientific (A11032)	Goat	2 µg/mL (IF)

## Supplementary Materials

The following are available online at https://www.mdpi.com/2073-4409/9/9/2137/s1, Table S1: Chemicals and kits used in this study, Table S2: Media components, Figure S1: KEGG and Reactome enrichment analysis from the RNA-sequencing performed on NCF hAECs, Figure S2: PANTHER enrichment pathway analysis of downregulated and upregulated genes in UNC- compared to SC-grown NCF hAECs, Table S3: Differential expression of genes of the Claudin family, Table S4: Differential expression of genes of the Monocarboxylate transporters (MCT) family, Table S5: Differential expression of genes of the Na+/H+ Exchangers and H+ channels families, Table S6: Differential expression of genes of the chaperones family.
