# Supplementary material for: Choice of Differentiation Media Significantly Impacts Cell Lineage and Response to CFTR Modulators in Fully Differentiated Primary Cultures of Cystic Fibrosis Human Airway Epithelial Cells
Source: Cells. 2020 Sep 21;9(9):2137. doi: 10.3390/cells9092137 (PMC7565948; doi:10.3390/cells9092137)
Supplement: Supplementary file 1 [file cells-09-02137-s001.zip › Table S1_proofs.pdf]

Table S1. Chemicals and kits used in this study

Chemical	Supplier	Catalog #
Albumin, Bovine, Fraction V, 98%	Alfa Aesar	J64100.22
Alcian Blue solution	Sigma-Aldrich	B8438
Amiloride hydrochloride hydrate	Sigma-Aldrich	A7410
BCA protein Assay	ThermoFisher Scientific	10678484
$CaCl_2$	Sigma-Aldrich	21115
CFTR corrector VX-809	CFF Therapeutics	
CFTR potentiator P5	CFF Therapeutics	
$CFTR_{inh172}$	Tocris Bioscence	3430
D-glucose	Sigma-Aldrich	G5767
Dextran-coupled Alexa Fluor 488	ThermoFisher Scientific	D22910
Dextran-coupled pHrodo Red	ThermoFisher Scientific	P10361
DMSO	Sigma-Aldrich	D2438
DPX Mountant for histology	Sigma-Aldrich	6522
Fetal bovine serum	Sigma-Aldrich	F7524
Forskolin	Tocris Bioscence	1099
Glycine	Sigma-Aldrich	G8898
Ham's F12 medium	ThermoFisher Scientific	11765054
Hematoxylin Solution, Harris Modified	Sigma-Aldrich	HHS16
Hepes sodium salt	Sigma-Aldrich	H3784
KCl	Sigma-Aldrich	P9541
MES	Sigma-Aldrich	M3885
Mitomycin C	Sigma-Aldrich	M4287
$MgCl_2$	Sigma-Aldrich	M1028
NaCl	Sigma-Aldrich	S9888
$NaHCO_3$	Sigma-Aldrich	S5761
Nigericin sodium salt	Sigma-Aldrich	N7143
PAS staining kit	Sigma-Aldrich	1.01646
PBS (phosphate buffer saline)	ThermoFisher Scientific	14190250
PFA (paraformaldehyde)	Sigma-Aldrich	P6148
pHrodo Red dextran	ThermoFisher Scientific	P10361
SDS (Sodium Dodecyl Sulfate)	Alfa Aesar	J63394
Sodium Deoxyxolate	Sigma-Aldrich	30970
Tris Base	Sigma-Aldrich	T1503
Triton X-100	Sigma-Aldrich	T8787
Tween-20	Sigma-Aldrich	P1379
UTP	Sigma-Aldrich	U6750
Xylene	Sigma-Aldrich	534056
Y-27632	Tocris Bioscence	1254
