# Supplementary material for: Choice of Differentiation Media Significantly Impacts Cell Lineage and Response to CFTR Modulators in Fully Differentiated Primary Cultures of Cystic Fibrosis Human Airway Epithelial Cells
Source: Cells. 2020 Sep 21;9(9):2137. doi: 10.3390/cells9092137 (PMC7565948; doi:10.3390/cells9092137)
Supplement: Supplementary file 1 [file cells-09-02137-s001.zip › Figures S1S2.pdf]

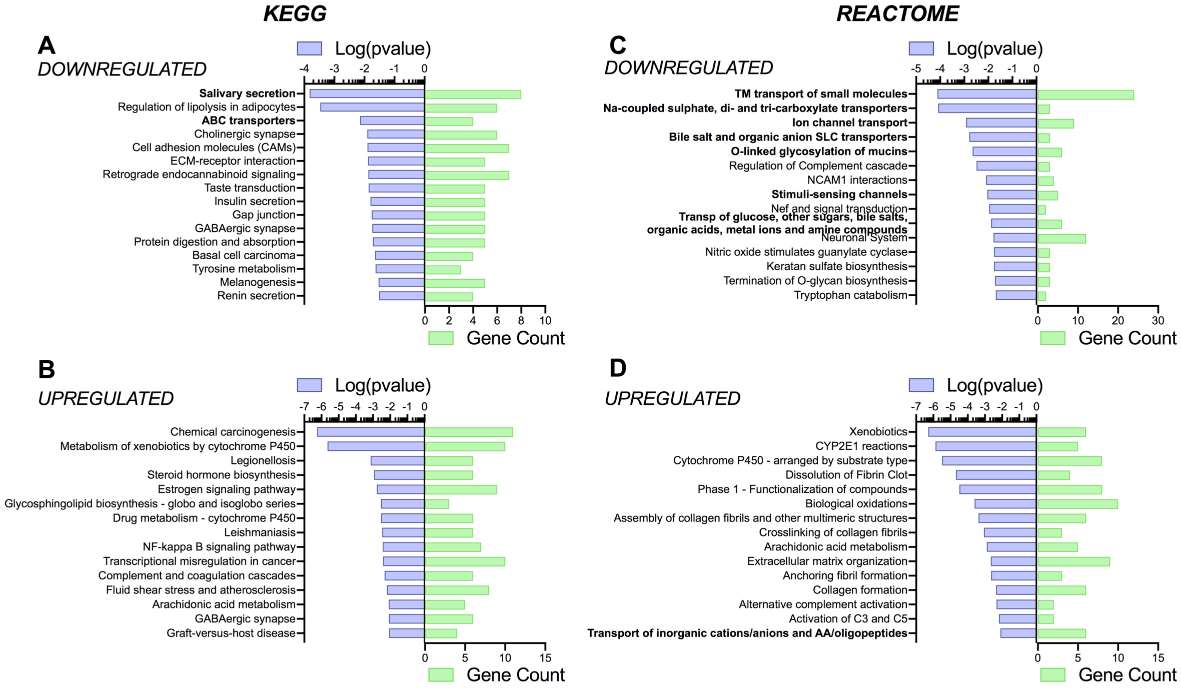

Figure S1. . KEGG (A,B), and Reactome (C,D) enrichment analysis from the RNA-sequencing performed on NCF hAECs. Top 15 pathways associated with downregulated (A,C) and upregulated (B,D) mRNAs in CF hAECs grow in UNC compared to SC media. Pathways identified are represented on the y-axis. The left x-axes correspond to the log of the P-value (purple) and the right x-axes correspond to the number of significant genes found in a given pathway (green).

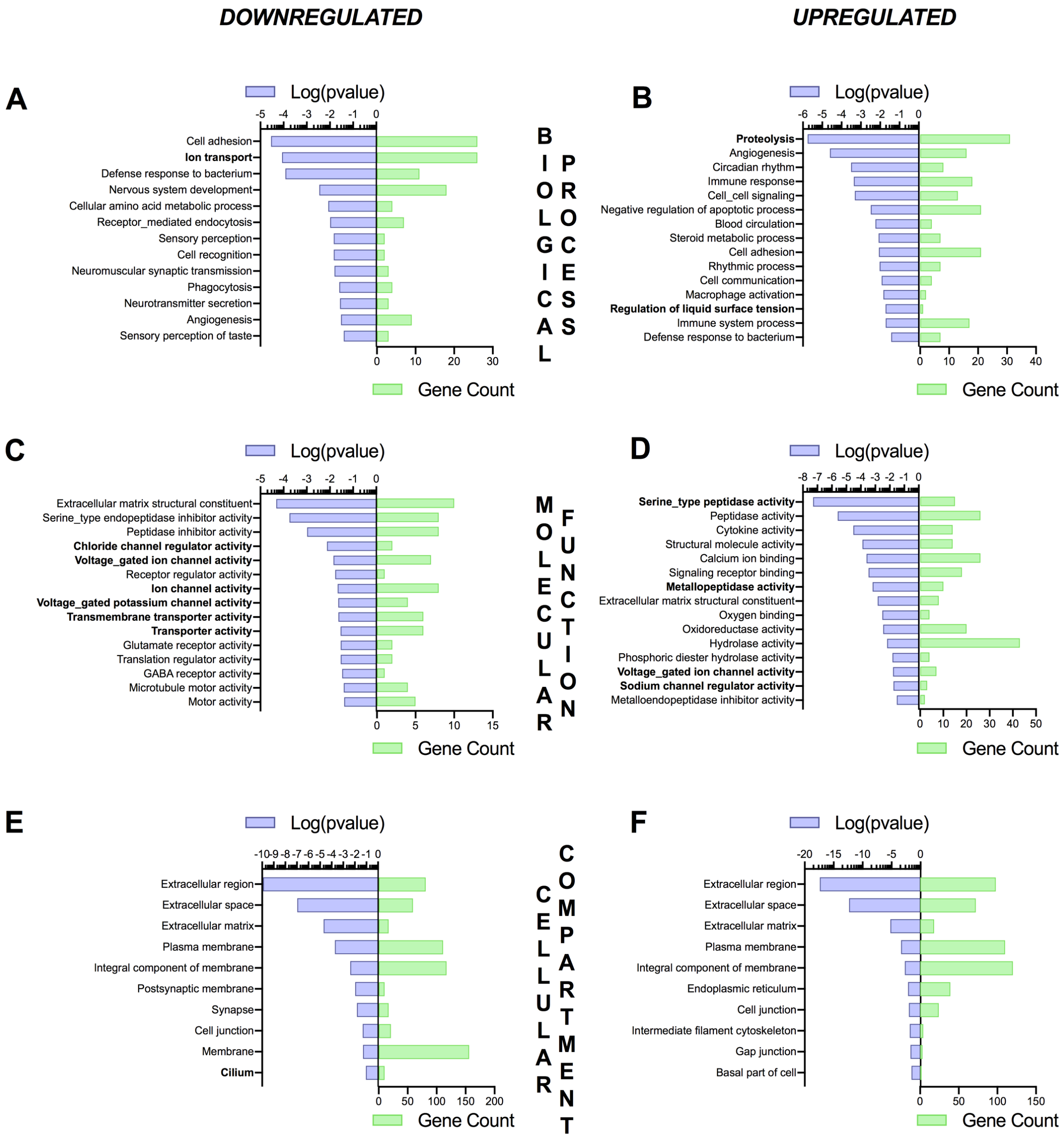

Figure S2. PANTHER enrichment pathway analysis of downregulated (A, C and E) and upregulated (B, D and F) genes in UNC compared to SC grown NCF hAECs. Top 10-15 items of biological process (A, B), molecular function (C, D) and cellular component (E, F) pathways using PANTHER database. Pathways identified are represented on the y-axis. The left x-axes correspond to the log of the P-value (purple) and the right x-axes correspond to the number of significant genes found in a given pathway (green).
