# Supplementary material for: Choice of Differentiation Media Significantly Impacts Cell Lineage and Response to CFTR Modulators in Fully Differentiated Primary Cultures of Cystic Fibrosis Human Airway Epithelial Cells
Source: Cells. 2020 Sep 21;9(9):2137. doi: 10.3390/cells9092137 (PMC7565948; doi:10.3390/cells9092137)
Supplement: Supplementary file 1 [file cells-09-02137-s001.zip › Table S3.pdf]

Table S3. Differential expression of genes of the Claudin family. ns: non significant

hgnc_symbol	Log_2FC	p-adj	Description		Result
CLDN1	-0.0741	7.56×10^{-01}	claudin 1 [Source:HGNC Symbol;Acc:HGNC:2032]	ns	
CLDN3	-1.2718	3.54×10^{-05}	claudin 3 [Source:HGNC Symbol;Acc:HGNC:2045]	significant	increased in SC
CLDN4	-0.0794	6.76×10^{-01}	claudin 4 [Source:HGNC Symbol;Acc:HGNC:2046]	ns	
CLDN5	3.2850	3.48×10^{-02}	claudin 5 [Source:HGNC Symbol;Acc:HGNC:2047]	significant	increased in UNC
CLDN7	1.4004	3.11×10^{-30}	claudin 7 [Source:HGNC Symbol;Acc:HGNC:2049]	significant	increased in UNC
CLDN8	1.1198	1.06×10^{-09}	claudin 8 [Source:HGNC Symbol;Acc:HGNC:2050]	significant	increased in UNC
CLDN9	-0.7062	8.82×10^{-02}	claudin 9 [Source:HGNC Symbol;Acc:HGNC:2051]	ns	
CLDN10	-2.4751	1.78×10^{-21}	claudin 10 [Source:HGNC Symbol;Acc:HGNC:2033]	significant	increased in SC
CLDN11	1.5002	2.95×10^{-01}	claudin 11 [Source:HGNC Symbol;Acc:HGNC:8514]	ns	
CLDN12	-0.0842	7.21×10^{-01}	claudin 12 [Source:HGNC Symbol;Acc:HGNC:2034]	ns	
CLDN15	-0.0431	9.47×10^{-01}	claudin 15 [Source:HGNC Symbol;Acc:HGNC:2036]	ns	
CLDN16	-1.1784	2.02×10^{-07}	claudin 16 [Source:HGNC Symbol;Acc:HGNC:2037]	significant	increased in SC
CLDN17	3.3118	1.30×10^{-01}	claudin 17 [Source:HGNC Symbol;Acc:HGNC:2038]	ns	
CLDN18	0.2530	8.75×10^{-01}	claudin 18 [Source:HGNC Symbol;Acc:HGNC:2039]	ns	
CLDN19	-2.4860	2.31×10^{-02}	claudin 19 [Source:HGNC Symbol;Acc:HGNC:2040]	significant	increased in SC
CLDN22	0.0271	9.62×10^{-01}	claudin 22 [Source:HGNC Symbol;Acc:HGNC:2044]	ns	
CLDN23	0.0703	8.96×10^{-01}	claudin 23 [Source:HGNC Symbol;Acc:HGNC:17591]	ns	
CLDN24	-2.7158	2.00×10^{-01}	claudin 24 [Source:HGNC Symbol;Acc:HGNC:37200]	ns	
