# Supplementary material for: Choice of Differentiation Media Significantly Impacts Cell Lineage and Response to CFTR Modulators in Fully Differentiated Primary Cultures of Cystic Fibrosis Human Airway Epithelial Cells
Source: Cells. 2020 Sep 21;9(9):2137. doi: 10.3390/cells9092137 (PMC7565948; doi:10.3390/cells9092137)
Supplement: Supplementary file 1 [file cells-09-02137-s001.zip › Table S4.pdf]

Table S4. Differential expression of genes of the Monocarboxylate transporters (MCT) family. ns: non significant

hgnc_symbol	Synonym	Log_2FC	p-adj	Description	Result	
SLC16A1	MCT1	1.5083	2.54×10^{-17}	solute carrier family 16 member 1 [Source:HGNC Symbol;Acc:HGNC:10922]	significant	increased in UNC
SLC16A2	MCT8	1.0206	3.25×10^{-06}	solute carrier family 16 member 2 [Source:HGNC Symbol;Acc:HGNC:10923]	significant	increased in UNC
SLC16A3	MCT4	1.0920	8.35×10^{-10}	solute carrier family 16 member 3 [Source:HGNC Symbol;Acc:HGNC:10924]	significant	increased in UNC
SLC16A4	MCT5	1.4438	3.53×10^{-15}	solute carrier family 16 member 4 [Source:HGNC Symbol;Acc:HGNC:10925]	significant	increased in UNC
SLC16A5	MCT6	-1.2266	8.09×10^{-15}	solute carrier family 16 member 5 [Source:HGNC Symbol;Acc:HGNC:10926]	significant	increased in SC
SLC16A6	MCT7	2.4929	1.18×10^{-06}	solute carrier family 16 member 6 [Source:HGNC Symbol;Acc:HGNC:10927]	significant	increased in UNC
SLC16A7	MCT2	0.7559	1.02×10^{-03}	solute carrier family 16 member 7 [Source:HGNC Symbol;Acc:HGNC:10928]	less than 2 fold change	
SLC16A9	MCT9	0.6411	3.03×10^{-04}	solute carrier family 16 member 9 [Source:HGNC Symbol;Acc:HGNC:23520]	less than 2 fold change	
SLC16A10	MCT10	-0.1009	9.54×10^{-01}	solute carrier family 16 member 10 [Source:HGNC Symbol;Acc:HGNC:17027]	ns	
SLC16A11	MCT11	1.6354	5.88×10^{-04}	solute carrier family 16 member 11 [Source:HGNC Symbol;Acc:HGNC:23093]	significant	increased in UNC
SLC16A12	MCT12	1.3885	1.93×10^{-01}	solute carrier family 16 member 12 [Source:HGNC Symbol;Acc:HGNC:23094]	ns	
SLC16A13	MCT13	0.2453	4.16×10^{-01}	solute carrier family 16 member 13 [Source:HGNC Symbol;Acc:HGNC:31037]	ns	
SLC16A14	MCT14	1.1117	4.89×10^{-06}	solute carrier family 16 member 14 [Source:HGNC Symbol;Acc:HGNC:26417]	significant	increased in UNC
