# Supplementary material for: Choice of Differentiation Media Significantly Impacts Cell Lineage and Response to CFTR Modulators in Fully Differentiated Primary Cultures of Cystic Fibrosis Human Airway Epithelial Cells
Source: Cells. 2020 Sep 21;9(9):2137. doi: 10.3390/cells9092137 (PMC7565948; doi:10.3390/cells9092137)
Supplement: Supplementary file 1 [file cells-09-02137-s001.zip › Table S5.pdf]

Table S5. Differential expression of genes of the Na^+/H^+ Exchangers and H^+ channels families. ns: non significant

hgnc_symbol	Log_2FC	p-adj	Description	Result	
Na^+ / H^+ Exchangers					
SLC9A1	0.979	5.29×10^{-14}	solute carrier family 9 member A1 [Source:HGNC Symbol;Acc:HGNC:11071]	less than 2 fold change	
SLC9A2	2.580	8.29×10^{-07}	solute carrier family 9 member A2 [Source:HGNC Symbol;Acc:HGNC:11072]	significant	increased in UNC
SLC9A3	-0.554	5.88×10^{-02}	solute carrier family 9 member A3 [Source:HGNC Symbol;Acc:HGNC:11073]	ns	
SLC9A4	2.582	4.22×10^{-12}	solute carrier family 9 member A4 [Source:HGNC Symbol;Acc:HGNC:11077]	significant	increased in UNC
SLC9A5	0.983	5.74×10^{-01}	solute carrier family 9 member A5 [Source:HGNC Symbol;Acc:HGNC:11078]	ns	
SLC9A6	0.590	9.71×10^{-02}	solute carrier family 9 member A6 [Source:HGNC Symbol;Acc:HGNC:11079]	ns	
SLC9A7	0.778	1.24×10^{-02}	solute carrier family 9 member A7 [Source:HGNC Symbol;Acc:HGNC:17123]	less than 2 fold change	
SLC9A8	0.687	3.15×10^{-07}	solute carrier family 9 member A8 [Source:HGNC Symbol;Acc:HGNC:20728]	less than 2 fold change	
SLC9A9	-1.368	3.79×10^{-04}	solute carrier family 9 member A9 [Source:HGNC Symbol;Acc:HGNC:20653]	significant	increased in SC
H^+ channels					
ATP6V1G3	-2.895	7.31×10^{-02}	ATPase H+ transporting V1 subunit G3 [Source:HGNC Symbol;Acc:HGNC:18265]	non significant	
ATP6V1B1	1.592	1.06×10^{-04}	ATPase H+ transporting V1 subunit B1 [Source:HGNC Symbol;Acc:HGNC:853]	significant	increased in UNC
ATP6V0A4	-1.529	1.61×10^{-08}	ATPase H+ transporting V0 subunit a4 [Source:HGNC Symbol;Acc:HGNC:866]	significant	increased in SC
ATP6V0E2	0.687	4.20×10^{-04}	ATPase H+ transporting V0 subunit e2 [Source:HGNC Symbol;Acc:HGNC:21723]	less than 2 fold change	
ATP6V0B	0.681	6.01×10^{-08}	ATPase H+ transporting V0 subunit b [Source:HGNC Symbol;Acc:HGNC:861]	less than 2 fold change	
ATP6V0E1	0.672	2.26×10^{-11}	ATPase H+ transporting V0 subunit e1 [Source:HGNC Symbol;Acc:HGNC:863]	less than 2 fold change	
ATP6V1E1	0.572	1.24×10^{-08}	ATPase H+ transporting V1 subunit E1 [Source:HGNC Symbol;Acc:HGNC:857]	less than 2 fold change	
ATP6V1C2	-0.473	9.34×10^{-02}	ATPase H+ transporting V1 subunit C2 [Source:HGNC Symbol;Acc:HGNC:18264]	less than 2 fold change	
ATP6V1B2	0.441	1.48×10^{-03}	ATPase H+ transporting V1 subunit B2 [Source:HGNC Symbol;Acc:HGNC:854]	less than 2 fold change	
ATP6V1C1	0.391	2.95×10^{-02}	ATPase H+ transporting V1 subunit C1 [Source:HGNC Symbol;Acc:HGNC:856]	less than 2 fold change	
ATP6V1F	0.356	1.02×10^{-02}	ATPase H+ transporting V1 subunit F [Source:HGNC Symbol;Acc:HGNC:16832]	less than 2 fold change	
ATP6V1H	0.334	1.84×10^{-02}	ATPase H+ transporting V1 subunit H [Source:HGNC Symbol;Acc:HGNC:18303]	less than 2 fold change	
ATP6V1A	0.295	2.48×10^{-02}	ATPase H+ transporting V1 subunit A [Source:HGNC Symbol;Acc:HGNC:851]	less than 2 fold change	
ATP6V1G2	-0.264	8.88×10^{-01}	ATPase H+ transporting V1 subunit G2	less than 2 fold change	

			[Source:HGNC Symbol;Acc:HGNC:862]	
ATP6V0A2	0.234	2.92×10^{-01}	ATPase H+ transporting V0 subunit a2 [Source:HGNC Symbol;Acc:HGNC:18481]	less than 2 fold change
ATP6V0D1	0.234	1.15×10^{-01}	ATPase H+ transporting V0 subunit d1 [Source:HGNC Symbol;Acc:HGNC:13724]	less than 2 fold change
ATP6V0C	0.221	1.93×10^{-01}	ATPase H+ transporting V0 subunit c [Source:HGNC Symbol;Acc:HGNC:855]	less than 2 fold change
ATP6AP2	0.181	1.62×10^{-01}	ATPase H+ transporting accessory protein 2 [Source:HGNC Symbol;Acc:HGNC:18305]	less than 2 fold change
ATP6V0D2	-0.173	9.21×10^{-01}	ATPase H+ transporting V0 subunit d2 [Source:HGNC Symbol;Acc:HGNC:18266]	less than 2 fold change
ATP6V1G1	0.167	2.18×10^{-01}	ATPase H+ transporting V1 subunit G1 [Source:HGNC Symbol;Acc:HGNC:864]	less than 2 fold change
ATP6V1D	-0.165	3.34×10^{-01}	ATPase H+ transporting V1 subunit D [Source:HGNC Symbol;Acc:HGNC:13527]	less than 2 fold change
ATP6V0A1	0.076	8.24×10^{-01}	ATPase H+ transporting V0 subunit a1 [Source:HGNC Symbol;Acc:HGNC:865]	less than 2 fold change
ATP6V1E2	0.061	9.02×10^{-01}	ATPase H+ transporting V1 subunit E2 [Source:HGNC Symbol;Acc:HGNC:18125]	less than 2 fold change
