# Supplementary material for: Choice of Differentiation Media Significantly Impacts Cell Lineage and Response to CFTR Modulators in Fully Differentiated Primary Cultures of Cystic Fibrosis Human Airway Epithelial Cells
Source: Cells. 2020 Sep 21;9(9):2137. doi: 10.3390/cells9092137 (PMC7565948; doi:10.3390/cells9092137)
Supplement: Supplementary file 1 [file cells-09-02137-s001.zip › Table S6.pdf]

Table S6. Differential expression of genes of the chaperones family. Genes in italics are known to interact with CFTR; ns: non significant.

hgnc_symbol	Synonym	Log_2FC	p-adj	Description	Result	
HSPA1A	*HSP70 member 1A*	*-0.618*	*3.27×10^{-03}*	*heat shock protein family A (Hsp70) member 1A [Source:HGNC Symbol;Acc:HGNC:5232]*	less than 2 fold change	
HSPA1B	*HSP70 member 1B*	*-0.287*	*1.75×10^{-01}*	*heat shock protein family A (Hsp70) member 1B [Source:HGNC Symbol;Acc:HGNC:5233]*	ns	
HSPA2	HSP70 member 2	-1.182	4.06×10^{-04}	heat shock protein family A (Hsp70) member 2 [Source:HGNC Symbol;Acc:HGNC:5235]	significant	increased in SC
HSPA4	HSP70 member 4	0.202	7.67×10^{-02}	heat shock protein family A (Hsp70) member 4 [Source:HGNC Symbol;Acc:HGNC:5237]	ns	
HSPA5	HSP70 member 5	0.845	1.97×10^{-20}	heat shock protein family A (Hsp70) member 5 [Source:HGNC Symbol;Acc:HGNC:5238]	less than 2 fold change	
HSPA6	HSP70-B′	1.668	6.58×10^{-02}	heat shock protein family A (Hsp70) member 6 [Source:HGNC Symbol;Acc:HGNC:5239]	ns	
HSPA8	*HSC70*	*-0.137*	*3.63×10^{-01}*	*heat shock protein family A (Hsp70) member 8 [Source:HGNC Symbol;Acc:HGNC:5241]*	ns	
HSPA9	HSP70 member 9	0.058	7.53×10^{-01}	heat shock protein family A (Hsp70) member 9 [Source:HGNC Symbol;Acc:HGNC:5244]	ns	
HSPA12A	HSP70 member 12A	-0.726	1.38×10^{-02}	heat shock protein family A (Hsp70) member 12A [Source:HGNC Symbol;Acc:HGNC:19022]	less than 2 fold change	
HSPA13	HSP70 member 13	0.680	1.68×10^{-04}	heat shock protein family A (Hsp70) member 13 [Source:HGNC Symbol;Acc:HGNC:11375]	less than 2 fold change	
HSPA14	HSP70 member 14	0.345	6.18×10^{-02}	heat shock protein family A (Hsp70) member 14 [Source:HGNC Symbol;Acc:HGNC:29526]	ns	
HSPB1	small HSP member 1	-0.084	7.21×10^{-01}	heat shock protein family B (small) member 1 [Source:HGNC Symbol;Acc:HGNC:5246]	ns	
HSPB8	small HSP member 8	0.473	2.50×10^{-01}	heat shock protein family B (small) member 8 [Source:HGNC Symbol;Acc:HGNC:30171]	ns	
HSPB11	small HSP member 11	-0.382	5.64×10^{-03}	heat shock protein family B (small) member 11 [Source:HGNC Symbol;Acc:HGNC:25019]	less than 2 fold change	
HSPD1	HSP60 member 1	-0.330	1.69×10^{-02}	heat shock protein family D (Hsp60) member 1 [Source:HGNC Symbol;Acc:HGNC:5261]	less than 2 fold change	
HSPE1	HSP10 member 1	-0.327	1.14×10^{-02}	heat shock protein family E (Hsp10) member 1 [Source:HGNC Symbol;Acc:HGNC:5269]	less than 2 fold change	
HSP90AA1	*HSP90AA1*	*-0.699*	*6.14×10^{-06}*	*heat shock protein 90 alpha family class A member 1 [Source:HGNC Symbol;Acc:HGNC:5253]*	less than 2 fold change	
HSP90AB1	HSP90AB1	-0.396	1.19×10^{-04}	heat shock protein 90 alpha family class B member 1 [Source:HGNC Symbol;Acc:HGNC:5258]	less than 2 fold change	

HSP90B1	HSP90B1	0.383	$2.65{\times}10^{-03}$	heat shock protein 90 beta family member 1 [Source:HGNC Symbol;Acc:HGNC:12028]	less than 2 fold change	
**Co-chaperones**						
HSPH1	*HSP110 member 1*	*-0.915*	*$1.69{\times}10^{-11}$*	*heat shock protein family H (Hsp110) member 1 [Source:HGNC Symbol;Acc:HGNC:16969]*	less than 2 fold change	
DNAJ co-chaperones						
DNAJA1		*-0.192*	*$9.06{\times}10^{-02}$*	*DnaJ heat shock protein family (Hsp40) member A1 [Source:HGNC Symbol;Acc:HGNC:5229]*	ns	
DNAJA2		0.311	$3.45{\times}10^{-03}$	DnaJ heat shock protein family (Hsp40) member A2 [Source:HGNC Symbol;Acc:HGNC:14884]	less than 2 fold change	
DNAJA3		0.137	$4.32{\times}10^{-01}$	DnaJ heat shock protein family (Hsp40) member A3 [Source:HGNC Symbol;Acc:HGNC:11808]	ns	
DNAJA4		-0.817	$7.16{\times}10^{-04}$	DnaJ heat shock protein family (Hsp40) member A4 [Source:HGNC Symbol;Acc:HGNC:14885]	less than 2 fold change	
DNAJB1		*0.032*	*$8.85{\times}10^{-01}$*	*DnaJ heat shock protein family (Hsp40) member B1 [Source:HGNC Symbol;Acc:HGNC:5270]*	ns	
DNAJB2		*-0.238*	*$1.65{\times}10^{-01}$*	*DnaJ heat shock protein family (Hsp40) member B2 [Source:HGNC Symbol;Acc:HGNC:5228]*	ns	
DNAJB4		-0.053	$8.30{\times}10^{-01}$	DnaJ heat shock protein family (Hsp40) member B4 [Source:HGNC Symbol;Acc:HGNC:14886]	ns	
DNAJB5		1.543	$1.21{\times}10^{-08}$	DnaJ heat shock protein family (Hsp40) member B5 [Source:HGNC Symbol;Acc:HGNC:14887]	significant	increased in UNC
DNAJB6		-0.204	$2.29{\times}10^{-01}$	DnaJ heat shock protein family (Hsp40) member B6 [Source:HGNC Symbol;Acc:HGNC:14888]	ns	
DNAJB9		0.745	$1.28{\times}10^{-03}$	DnaJ heat shock protein family (Hsp40) member B9 [Source:HGNC Symbol;Acc:HGNC:6968]	less than 2 fold change	
DNAJB11		0.403	$4.09{\times}10^{-03}$	DnaJ heat shock protein family (Hsp40) member B11 [Source:HGNC Symbol;Acc:HGNC:14889]	less than 2 fold change	
DNAJB12		*0.177*	*$3.31{\times}10^{-01}$*	*DnaJ heat shock protein family (Hsp40) member B12 [Source:HGNC Symbol;Acc:HGNC:14891]*	ns	
DNAJB13		-0.984	$1.23{\times}10^{-08}$	DnaJ heat shock protein family (Hsp40) member B13 [Source:HGNC Symbol;Acc:HGNC:30718]	less than 2 fold change	
DNAJB14		0.197	$3.76{\times}10^{-01}$	DnaJ heat shock protein family (Hsp40) member B14 [Source:HGNC Symbol;Acc:HGNC:25881]	ns	
DNAJC1		0.955	$1.64{\times}10^{-07}$	DnaJ heat shock protein family (Hsp40) member C1 [Source:HGNC Symbol;Acc:HGNC:20090]	less than 2 fold change	
DNAJC2		-0.091	$6.44{\times}10^{-01}$	DnaJ heat shock protein family (Hsp40) member C2 [Source:HGNC Symbol;Acc:HGNC:13192]	ns	
DNAJC3		0.356	$4.27{\times}10^{-03}$	DnaJ heat shock protein family (Hsp40) member C3 [Source:HGNC Symbol;Acc:HGNC:9439]	less than 2 fold change	

DNAJC4	0.067	8.33×10^{-01}	DnaJ heat shock protein family (Hsp40) member C4 [Source:HGNC Symbol;Acc:HGNC:5271]	ns	
DNAJC5	*0.298*	*5.12×10^{-03}*	*DnaJ heat shock protein family (Hsp40) member C5 [Source:HGNC Symbol;Acc:HGNC:16235]*	less than 2 fold change	
DNAJC5B	-2.420	4.39×10^{-02}	DnaJ heat shock protein family (Hsp40) member C5 beta [Source:HGNC Symbol;Acc:HGNC:24138]	significant	increased in SC
DNAJC6	-0.766	7.30×10^{-02}	DnaJ heat shock protein family (Hsp40) member C6 [Source:HGNC Symbol;Acc:HGNC:15469]	ns	
DNAJC7	*0.171*	*2.04×10^{-01}*	*DnaJ heat shock protein family (Hsp40) member C7 [Source:HGNC Symbol;Acc:HGNC:12392]*	ns	
DNAJC8	0.163	4.37×10^{-01}	DnaJ heat shock protein family (Hsp40) member C8 [Source:HGNC Symbol;Acc:HGNC:15470]	ns	
DNAJC9	-0.015	9.69×10^{-01}	DnaJ heat shock protein family (Hsp40) member C9 [Source:HGNC Symbol;Acc:HGNC:19123]	ns	
DNAJC10	-0.165	3.62×10^{-01}	DnaJ heat shock protein family (Hsp40) member C10 [Source:HGNC Symbol;Acc:HGNC:24637]	ns	
DNAJC11	0.095	6.81×10^{-01}	DnaJ heat shock protein family (Hsp40) member C11 [Source:HGNC Symbol;Acc:HGNC:25570]	ns	
DNAJC12	2.043	9.30×10^{-05}	DnaJ heat shock protein family (Hsp40) member C12 [Source:HGNC Symbol;Acc:HGNC:28908]	significant	increased in UNC
DNAJC13	0.399	3.96×10^{-02}	DnaJ heat shock protein family (Hsp40) member C13 [Source:HGNC Symbol;Acc:HGNC:30343]	less than 2 fold change	
DNAJC14	0.017	9.41×10^{-01}	DnaJ heat shock protein family (Hsp40) member C14 [Source:HGNC Symbol;Acc:HGNC:24581]	ns	
DNAJC15	0.476	1.74×10^{-03}	DnaJ heat shock protein family (Hsp40) member C15 [Source:HGNC Symbol;Acc:HGNC:20325]	less than 2 fold change	
DNAJC16	0.159	5.46×10^{-01}	DnaJ heat shock protein family (Hsp40) member C16 [Source:HGNC Symbol;Acc:HGNC:29157]	ns	
DNAJC17	0.015	9.68×10^{-01}	DnaJ heat shock protein family (Hsp40) member C17 [Source:HGNC Symbol;Acc:HGNC:25556]	ns	
DNAJC18	0.022	9.66×10^{-01}	DnaJ heat shock protein family (Hsp40) member C18 [Source:HGNC Symbol;Acc:HGNC:28429]	ns	
DNAJC19	0.021	9.41×10^{-01}	DnaJ heat shock protein family (Hsp40) member C19 [Source:HGNC Symbol;Acc:HGNC:30528]	ns	
DNAJC21	0.252	1.65×10^{-01}	DnaJ heat shock protein family (Hsp40) member C21 [Source:HGNC Symbol;Acc:HGNC:27030]	ns	
DNAJC22	-1.714	4.29×10^{-07}	DnaJ heat shock protein family (Hsp40)	significant	increased

			member C22 [Source:HGNC Symbol;Acc:HGNC:25802]		in SC
DNAJC24	0.254	6.22×10^{-01}	DnaJ heat shock protein family (Hsp40) member C24 [Source:HGNC Symbol;Acc:HGNC:26979]	ns	
DNAJC25	0.371	1.45×10^{-01}	DnaJ heat shock protein family (Hsp40) member C25 [Source:HGNC Symbol;Acc:HGNC:34187]	ns	
DNAJC27	-0.632	6.11×10^{-02}	DnaJ heat shock protein family (Hsp40) member C27 [Source:HGNC Symbol;Acc:HGNC:30290]	ns	
DNAJC28	0.248	6.77×10^{-01}	DnaJ heat shock protein family (Hsp40) member C28 [Source:HGNC Symbol;Acc:HGNC:1297]	ns	
DNAJC30	0.076	8.19×10^{-01}	DnaJ heat shock protein family (Hsp40) member C30 [Source:HGNC Symbol;Acc:HGNC:16410]	ns	
BAG co-chaperones					
BAG1	*0.484*	*9.40×10^{-05}*	*BCL2 associated athanogene 1 [Source:HGNC Symbol;Acc:HGNC:937]*	less than 2 fold change	
BAG2	*-1.104*	*1.82×10^{-06}*	*BCL2 associated athanogene 2 [Source:HGNC Symbol;Acc:HGNC:938]*	significant	increased in SC
BAG3	0.124	3.63×10^{-01}	BCL2 associated athanogene 3 [Source:HGNC Symbol;Acc:HGNC:939]	ns	
BAG4	0.408	8.06×10^{-02}	BCL2 associated athanogene 4 [Source:HGNC Symbol;Acc:HGNC:940]	ns	
BAG5	-0.115	4.70×10^{-01}	BCL2 associated athanogene 5 [Source:HGNC Symbol;Acc:HGNC:941]	ns	
BAG6	0.099	5.46×10^{-01}	BCL2 associated athanogene 6 [Source:HGNC Symbol;Acc:HGNC:13919]	ns	
HSPBP1	*-0.810*	*1.44×10^{-09}*	*HSPA (Hsp70) binding protein 1 [Source:HGNC Symbol;Acc:HGNC:24989]*	less than 2 fold change	
STUB1	*-0.212*	*2.14×10^{-01}*	*STIP1 homology and U-box containing protein 1 [Source:HGNC Symbol;Acc:HGNC:11427]*	ns	
STIP1	*-0.117*	*5.35×10^{-01}*	*stress induced phosphoprotein 1 [Source:HGNC Symbol;Acc:HGNC:11387]*	ns	
