# Supplementary material for: Choice of Differentiation Media Significantly Impacts Cell Lineage and Response to CFTR Modulators in Fully Differentiated Primary Cultures of Cystic Fibrosis Human Airway Epithelial Cells
Source: Cells. 2020 Sep 21;9(9):2137. doi: 10.3390/cells9092137 (PMC7565948; doi:10.3390/cells9092137)
Supplement: Supplementary file 1 [file cells-09-02137-s001.zip › Table S2 proofs.pdf]

Table S2. Media components

Component	Supplier	Catalog #	Final concentration
**SC medium**			
PneumaCult-ALI Basal medium	STEMCELL Technologies	05002	
PneumaCult-ALI 10x Supplement	STEMCELL Technologies	05003	
Hydrocortisone Stock Solution	STEMCELL Technologies	07925	
PneumaCult-ALI Maintenance Supplement (100x)	STEMCELL Technologies	05006	
Heparin Solution	STEMCELL Technologies	07980	
Penicillin/Streptomycin	Thermo Fisher Scientific	15140-122	100 U/mL - 100 μg/mL
**BEGM / UNC**			
LHC Basal	Thermo Fisher Scientific	12677019	100 % - BEGM
DMEM high glucose	Thermo Fisher Scientific	11965-092	
LHC Basal : DMEM high glucose			50:50 - UNC
Bovine serum albumin	Sigma-Aldrich	A7638	0.5 mg/mL
Insulin	Sigma-Aldrich	I6634	8.7×10^{-7} M
Hydrocortisone	Sigma-Aldrich	H0396	2.1×10^{-7} M
Epidermal growth factor (EGF)	Thermo Fisher Scientific	PHG0313	25 ng/mL - BEGM<br>0.50 ng/mL - UNC
Triiodothyronine (T3)	Sigma-Aldrich	T6397	1×10^{-8} M
Transferrin	Sigma-Aldrich	T0665	1.25×10^{-7} M
Epinephrine	Sigma-Aldrich	E4250	2.7×10^{-6} M
Phosphoryl ethanolamine (PEA)	Sigma-Aldrich	P0503	5×10^{-7} M
Ethanolamine	Sigma-Aldrich	E0135	5×10^{-7} M
Bovine pituitary extract (BPE)	Sigma-Aldrich	P1476	10 μg/mL
Selenium	Sigma-Aldrich	S5261	3×10^{-8} M
Manganese	Sigma-Aldrich	M5005	10^{-9} M
Silicone	Sigma-Aldrich	S5904	5×10^{-7} M
Molybdenum	Sigma-Aldrich	M1019	10^{-9} M
Vanadium	Sigma-Aldrich	398128	5×10^{-9} M
Nickel sulphate	Sigma-Aldrich	N4882	10^{-9} M
Tin	Sigma-Aldrich	31669	5×10^{-10} M
Retinoic acid	Sigma-Aldrich	R2625	5×10^{-8} M
Zinc sulphate	Sigma-Aldrich	Z0251	3×10^{-6} M
Ferrous sulphate	Sigma-Aldrich	1270355	1.5×10^{-6} M
Magnesium chloride	Sigma-Aldrich	M2393	6×10^{-4} M
Calcium chloride	Sigma-Aldrich	C3881	1.1×10^{-4} M
Penicillin/Streptomycin	Thermo Fisher Scientific	15140-122	100 U/mL - 100 μg/mL – UNC only
Gentamicin	Sigma-Aldrich	G1397	50 μg/mL – BEGM only
Amphotericin B	Sigma-Aldrich	A2942	0.25 μg/mL – BEGM only
